# Construction of Interlayer Coupling Diatomic Nanozyme with Peroxidase‐Like and Photothermal Activities for Efficient Synergistic Antibacteria

**DOI:** 10.1002/advs.202305823

**Published:** 2024-03-09

**Authors:** Xiudong Shi, Jie Lv, Shuangling Deng, Fang Zhou, Jiangang Mei, Lei Zheng, Jing Zhang

**Affiliations:** ^1^ Department of Laboratory Medicine Nanfang Hospital Southern Medical University Guangzhou 510515 China

**Keywords:** antibacteria, interlayer coupling diatomic, nanozyme, photothermal activity

## Abstract

Pathogenic bacteria are the main cause of bacterial infectious diseases, which have posed a grave threat to public health. Single‐atom nanozymes have emerged as promising candidates for antibacterial applications, but their activities need to be further improved. Considering diatomic nanozymes exhibit superior metal loading capacities and enhanced catalytic performance, a new interlayer coupling diatomic nanozyme (IC‐DAN) is constructed by modulating the coordination environment in an atomic‐level engineering. It is well demonstrated that IC‐DAN exhibited superior peroxidase‐mimetic activity in the presence of H_2_O_2_ to yield abundant ∙OH and possessed high photothermal conversion ability, which synergistically achieves efficient antibacterial therapy. Therefore, IC‐DAN shows great potential used as antibacterial agent in clinic and this study open a new route to developing high‐performance artificial enzymes.

## Graphical abstract

A new interlayer coupling dual‐atom nanozyme is constructed with high‐efficiency catalytic performance and comparable kinetics. It can effectively catalyze the peroxidase‐like reaction to yield abundant ∙OH and possessed high‐efficiency photothermal conversion ability, which synergistically achieves efficient anti‐bacteria.

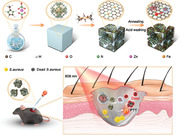

## 1 Introduction

Nanozymes are nanoscale materials that exhibit inherent enzyme‐mimetic properties. Recently, nanozymes have garnered significant interest for their ability to surmount the inherent constraints associated with natural enzymes, including elevated costs, reduced stability, and stringent storage conditions.^[^ ^1^, ^2^, ^3^, ^4^, ^5^ ^]^ Importantly, benefiting from their unique catalytic activities and physicochemical properties, nanozymes have exhibited great application potential.^[^ ^6^, ^7^, ^8^, ^9^, ^10^, ^11^, ^12^ ^]^ Despite their wide development, the application of nanozymes is markedly limited by their relatively low catalytic activity.^[^ ^3^, ^13^, ^14^, ^15^ ^]^ Catalysts consisting of single atoms, possessing precise electronic and geometric structures, could serve as advantageous substitutes for conventional enzymes. These catalysts possess the capacity to emulate the intricately evolved catalytic centers found in natural enzymes, operating at the atomic scale.

Despite considerable efforts, further breakthrough in the performance of single‐atom nanozymes faces an enormous challenge. To date, several research groups have explored diverse synthetic techniques to prepare single‐atom nanozymes for catalytic activity.^[^ ^3^, ^13^, ^14^, ^15^, ^16^, ^17^, ^18^, ^19^ ^]^ For instance, Liang's group designed a single‐atom nanozymes (SAN) with FeN_3_P as the center to regulate the electronic configuration of the single‐atom iron active site through the introduction of phosphorus coordination, which exhibited similar catalytic activity and enzyme kinetic properties to natural enzymes. In addition, it can significantly inhibit the growth of tumor cells.^[^ ^5^ ^]^ Huo's group prepared single‐atom iron nano‐catalyst SAF NCs by mixed pyrolysis of ZIF‐8 and ferric acetylacetone to produce abundant of hydroxyl radicals under acidic conditions to achieve tumor inhibition effect.^[^ ^6^, ^11^ ^]^ Liu et al. prepared highly active single‐atom nanozymes containing Zn‐porphyrin structures by using the strategy of mesoporous silicon protecting the precursor of ZIF‐8 to produce large quantities of hydroxyl radical from H_2_O_2_ to efficiently remove bacteria (such as *Staphylococcus aureus(S. aureus)* and *Escherichia coli (E. coli)*).^[^ ^9^ ^]^ Shi's team reported that Fe single‐atom nanozymes effectively induced peroxidase‐mimetic activity in the presence of H_2_O_2_, achieving substantial amounts of hydroxyl to efficiently remove bacteria such as *S. aureus* and *E. coli* to promote wound healing.^[^ ^7^ ^]^ Although single‐atom nanozymes show a broad spectrum of biomedical applications including antibacteria, there are still some problems that need to be addressed, such as insufficient diversity, relatively simple structure, and metal loading. Diatomic catalysts (DACs), as members of the monatomic catalytic family, have been emerging in recent years, and the cooperative interaction between two metal atoms (homonuclear/heteronuclear) in diatomic catalysis significantly improves the catalytic activity.^[^ ^20^, ^21^, ^22^ ^]^ In contrast to single‐atom nanozymes, diatomic nanozymes (DAN) offer not only the benefits of increased metal loading and enhanced atom utilization efficiency but also the capacity for synergistic collaboration among proximate metal atoms, enhances catalytic efficacy. Notably, the synthesized Fe_2_NC nanozyme demonstrated superior catalase (CAT)‐like, oxidase (OXD)‐like, and superoxide dismutase (SOD)‐like activities compared to its single‐atom counterpart, Fe_1_NC nanozyme.^[^ ^23^ ^]^ Accordingly, to solve the problems of single atom nanozyme mentioned above, constructing double‐atom structures with higher enzyme‐like activities is a good choice. However, preparing coupled diatomic nanozymes to achieve enhanced catalytic performance remains challenging, let alone the related antibacterial application.^[^ ^24^, ^25^ ^]^

**Scheme 1:**
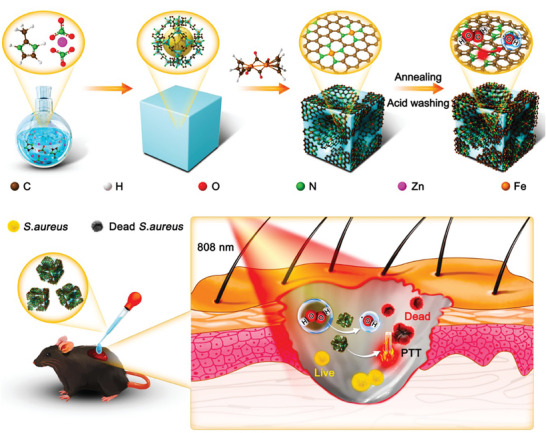
Construction of interlayer coupling diatomic nanozymes system and its application in rapid sterilization.

Herein, in this work, we intend to construct an interlayer coupling FeN_4_–FeN_4_ dual‐atom peroxidase nanozyme (IC‐DAN) by modulating the coordination environment of Fe in an atomic‐level engineered and explore its antibacterial performance. Ultimately, it was well demonstrated that IC‐DAN exhibited high‐efficiency catalytic performance and comparable kinetics. It proficiently catalyzes the peroxidase‐mimetic reaction, resulting in the prolific generation of ∙OH radicals, and possessed high‐efficiency photothermal conversion ability, which synergistically achieved efficient anti‐bacteria (**Scheme** **1**). Hence, this study could pave the way for the creation of artificial enzymes, emerging as highly promising alternatives to their natural enzymes.

## 2 Results and Discussion

### 2.1 Characterization of Nanozyme

The synthetic pathway to the FeN_4_–FeN_4_ dual‐atom peroxidase nanozyme (IC‐DAN) was delineated as Scheme 1. Initially, zeolitic imidazolate framework‐8 (ZIF‐8) nanocubes were synthesized via the previous literature,^[^ ^26^, ^27^ ^]^ employing cetyltrimethylammonium bromide (CTAB) as the surfactant, 2‐methylimidazole for ligand coordination, and zinc ions as nodal points. Subsequent to this, ZIF‐8 constructs underwent pyrolysis under an argon atmosphere, with cyclopentadienyliron dicarbonyl dimer introduced as a gaseous iron dopant. The dimer was evaporated to form a gas that permeated the ZIF‐8 nanocubes' porous architecture, undergoing decomposition to yield intricately curved nanostructures. This process facilitated the integration of paired iron atoms, precisely coordinated with nitrogen. The release of volatile carbon monoxide and dioxide gases facilitated the formation of a carbon material characterized by high curvature and a porous structure. Finally, the as‐targeted material (IC‐DAN) was subjected to a sulfuric acid treatment followed by an annealing process at 950 °C under an argon flux.

**Figure 1:**
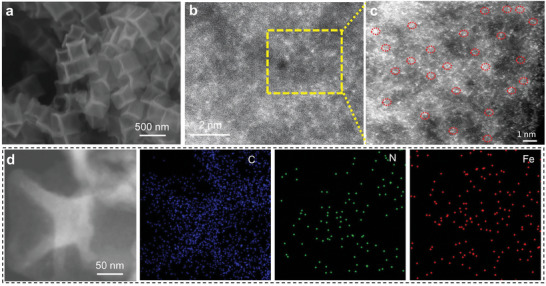
SEM image of a) IC‐DAN. b) STEM image of IC‐DAN. c) The magnified image of the partial area of the image. d) Elemental mapping of IC‐DAN.

The synthesized ZIF‐8 was elucidated using scanning electron microscopy (SEM), with representative images presented in Figure S1 (Supporting Information). The results indicated that N‐doped concave carbon (N‐CC) derived from the pyrolysis of ZIF‐8, and coupled iron‐doped ZIF‐8‐derived carbon (IC‐DAN) samples. The carbon material derived from ZIF‐8 (referred to as N‐CC) retained the well‐defined nanocubic morphology characteristic of its precursor, albeit showing notably smaller nanocubes (as depicted in Figure S2, Supporting Information). However, following the introduction of dual Fe atoms via the employment of cyclopentadienyliron dicarbonyl dimer as a gaseous iron dopant, a discernible transformation in the catalyst's morphology was observed. While the cubic morphology was largely preserved, each face of the cube exhibited a distinct concave feature, like the previously described single‐atom nanozymes (SAN) (Figure S3, Supporting Information). Importantly, IC‐DAN exhibited a notably larger size compared to both N‐CC and ZIF‐8 nanocubes, indicating that the incorporation of cyclopentadienyliron dicarbonyl dimer during the gaseous doping process instigated the expansion and consequential morphological alteration of the nanocubes (**Figure** **1**a), resulting in the formation of a more abundant porous architecture. Elemental mapping of IC‐DAN (Figure 1d) further provided evidence for the homogeneous dispersion of Fe and N atoms within the carbon framework derived from ZIF‐8. Furthermore, the scanning transmission electron microscope (STEM) image depicted in Figure 1b revealed the exceptional atomic‐scale dispersion of doped Fe within the material. No Fe nanoparticles could be observed, revealing that the gaseous doping strategy achieved remarkable Fe dispersion and incorporation. As depicted in the amplified image in Figure 1c, the iron (Fe) atoms, represented as bright dots, were uniformly distributed with coupled Fe atoms observable in every region at the atomic level. The distance between the two coupled Fe atoms was ≈0.26 nm, indicating that employing cyclopentadienyliron dicarbonyl dimer as a gaseous iron dopant realized the preparation of the concave‐face coupled Fe structure in the ultimately prepared ZIF‐8‐derived carbon material. Each Fe atom should bind with N atoms to establish the electronic configuration of “N_x_–Fe–Fe–N_x_”. Moreover, the results of X‐ray diffraction (XRD) analysis demonstrated the highly dispersed nature of the doped coupled iron atoms within the catalyst, with no discernible formation of Fe species particles (Figure S4, Supporting Information). The data of X‐ray photoelectron spectroscopy (XPS) and inductively coupled plasma optical emission spectrometer (ICP‐OES) also confirmed the high iron loading of IC‐DAN (Table S1, Supporting Information).

**Figure 2:**
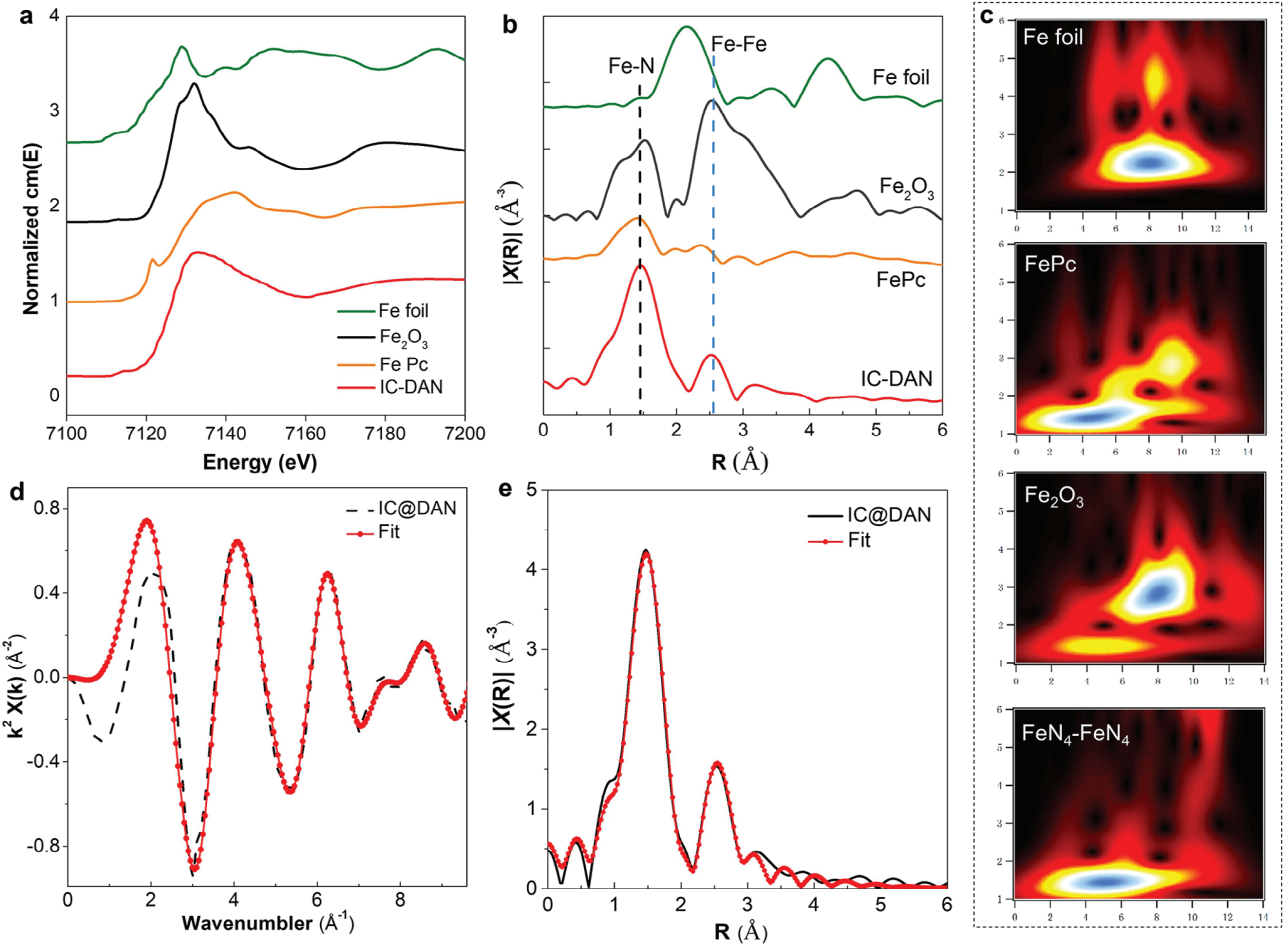
a) The experimental Fe K‐edge XANES spectra of IC‐DAN and reference samples. b) Fourier‐transformed magnitudes of the experimental Fe K‐edge EXAFS signals of IC‐DAN along with reference samples. c) Wavelet transforms (WT) spectra of Fe K‐edge for Fe foil, FePc, IC‐DAN (FeN_4_‐FeN_4_), and Fe_2_O_3_, respectively. d,e) Fitting curves of the EXAFS of IC‐DAN in the k‐space and r‐space.

In‐depth insights into the local atomic coordination of IC‐DAN were gained through the implementation of extended X‐ray absorption fine structure (EXAFS) analysis (**Figure** **2**b–e; Figures S6 and S7, Supporting Information) and X‐ray absorption spectroscopy (XAS) investigations (Figure 2a; Figure S5, Supporting Information).

The X‐ray absorption near edge structure (XANES) spectra at the Fe K‐edge, presented in Figure 2a, distinguish IC‐DAN from the comparative samples such as Fe foils and Fe_2_O_3_, aligning more closely with the spectra of SAN and FePc samples. This observation underscores the absence of Fe particles or oxides, highlighting the predominant coordination of Fe atoms with nitrogen within IC‐DAN. The EXAFS spectra findings in Figure S5 (Supporting Information) unveil a predominant peak at ≈1.5 Å, aligning with the Fe–N_4_ scattering path, a distinctive feature of SAN. Notably, the primary peak at ≈1.5 Å for SAN closely corresponds to the Fe–N_4_ scattering path, while the sharp fitting peak at a bond length of ≈2.6 Å for coupling diatomic iron was conspicuously absent. convincingly confirming the dispersion of Fe as solitary atoms within the N‐doped carbon matrix to form Fe–N_x_ entities. For IC‐DAN, apart from the main peak at ≈1.5 Å, an obvious coupling diatomic peak occurred at ≈2.6 Å, corroborating the presence of paired Fe atoms (Figure 2e). Taken all the results together, it could be concluded that our gaseous doping process successfully fabricated active sites with coupling diatomic iron.

In contrast, a discernible shift in the absorption edge of IC‐DAN toward lower energy relative to SAN (Figure S5, Supporting Information) signals the formation of coupling diatomic iron. Wavelet transform (WT) analysis of the Fe K‐edge EXAFS oscillations for both SAN and IC‐DAN, against references of Fe foil and FePc, are demonstrated in Figure 2c and Figure S10 (Supporting Information). SAN's WT contour plot displays a singular intensity peak at ≈5.0 Å^−1^, attributable to the Fe–N coordination in the first shell. Conversely, IC‐DAN manifests dual intensity peaks at ≈5.0 and 8.0 Å^−1^, indicative of Fe–N and Fe–Fe interactions in the first and second shells, respectively. The WT analysis manifests that SAN really exists as single Fe atoms while IC‐DAN shows augmented Fe–Fe bonding, aligning with EXAFS findings (Figure 2b; Figures S6 and S7, Supporting Information). The integration of XAS fitting results with WT analysis further specifies the coordination number of nitrogen to iron in IC‐DAN as approximately four (Figure 2e; Table S2, Supporting Information),^[^ ^28^, ^29^, ^30^, ^31^ ^]^ further implying that the coupled Fe atoms were present in the catalyst as N_4_–Fe–Fe–N_4_ active structures that resulted in greatly enhanced catalytic performance.

### 2.2 Theoretical Calculation

**Figure 3:**
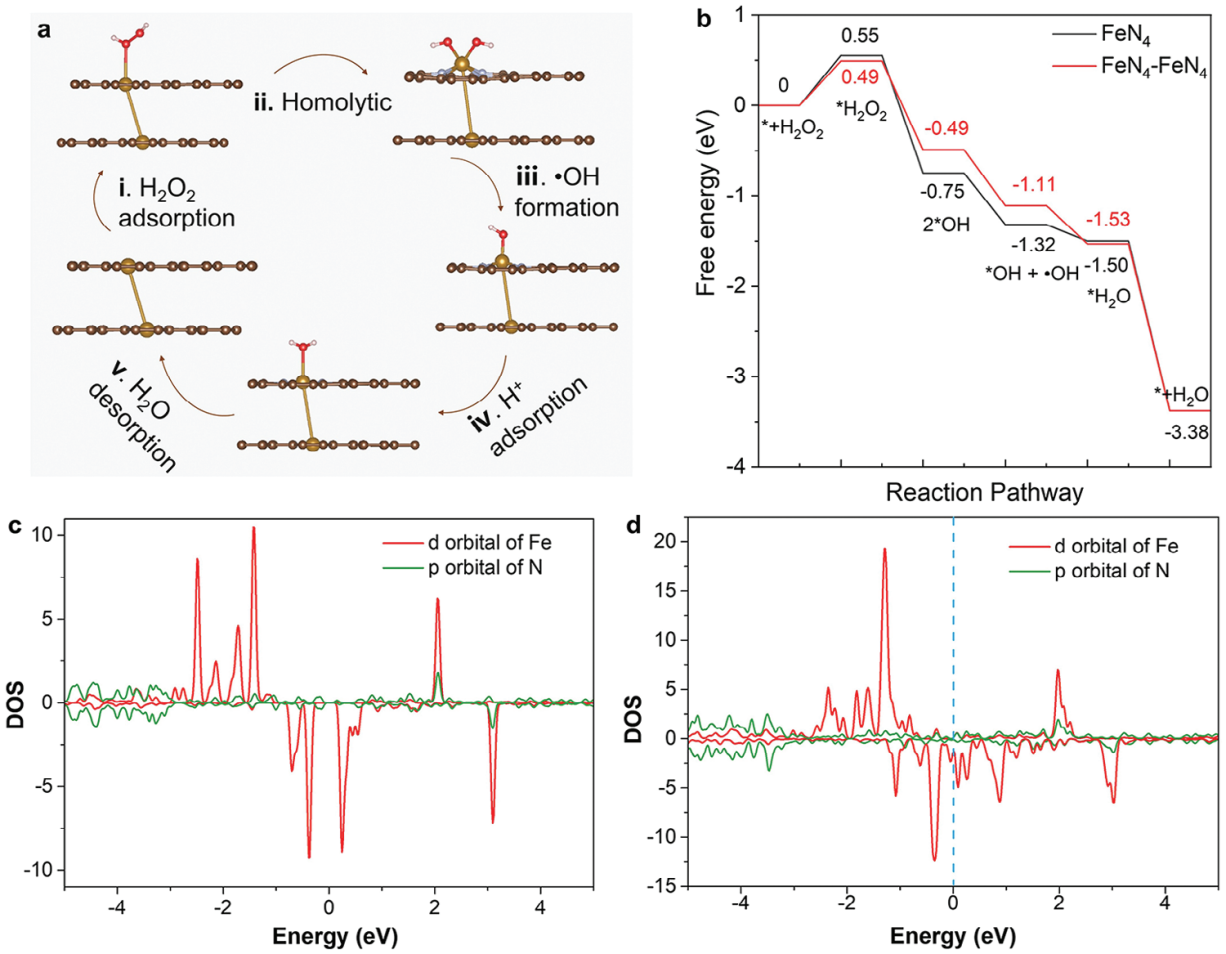
Theoretical calculations of the reaction process. a) Proposed catalytic mechanism for peroxidase‐like reaction on FeN_4_–FeN_4_. b) Corresponding free energy diagram for peroxidase‐like reaction on FeN_4_–FeN_4_ and FeN_4_. c) DOS of FeN_4_ SAzyme and d) FeN_4_–FeN_4_ DAzymes.

In order to better reveal the source of diatomic nanozymes peroxidase‐like activity, the DFT was calculated. The free energy diagram of diatomic peroxidase‐like activity and the energetics of key intermediates (O^*^, OH^*^, ∙OH, and OOH^*^) were elucidated through calculations based on density functional theory (DFT). Combined with previous studies on the partial structure of Fe–N_x_ active center, the optimized adsorption configuration of structure FeN_4_–FeN_4_ nanozymes is shown in **Figure** **3**a. The H_2_O_2_ adsorption configuration of structure FeN_4_–FeN_4_ nanozymes represents the initial phase of the subsequent mechanistic sequence. The adsorption energies of H_2_O_2_ adsorption on FeN_4_–FeN_4_ and FeN_4_ are 0.49 and 0.55 eV (Figure 3b), respectively. i) FeN_4_–FeN_4_ nanozymes were more likely to bind to H_2_O_2_ molecules (Figure S8, Supporting Information) due to the lower absorption energy involved. The reduced Fe─O bond length of 2.81 Å observed in the FeN_4_–FeN_4_ DAzyme, relative to that in the FeN_4_ SAzyme, evidences a higher affinity of FeN_4_–FeN_4_ DAzyme toward H_2_O_2_. Next, two ^*^OH species ii) are formed by homogeneous cleavage of adsorbed H_2_O_2_ molecules. Desorption iii) of ^*^OH at a single Fe site is the key step of the rate‐determining. The energy barrier of FeN_4_–FeN_4_ DAzyme registers significantly lower than that of FeN_4_ SAzyme (0.49 vs 0.75 eV), indicating that FeN_4_‐FeN_4_ DAzyme is toward the facilitation of ∙OH generation. During step iv), the acidic milieu promotes the association of ^*^OH with H^+^, culminating in the formation of ^*^H_2_O. Finally, both FeN_4_ SAzyme and FeN_4_–FeN_4_ DAzyme returned to their original state of molecular desorption of H_2_O (Figures S8 and S9, Supporting Information). As shown in Figure 3c,d, compared with FeN_4_ SAzyme, there are significantly higher density of states (DOS) when approaching the Fermi level, and more new hybrid electronic states of FeN_4_–FeN_4_ DAzyme can be observed, which proves stronger interaction and adsorption between FeN_4_–FeN_4_ DAzyme. And enhanced charge transfer during the peroxidase catalysis process.

### 2.3 Measurement of Catalytic Activity

**Figure 4:**
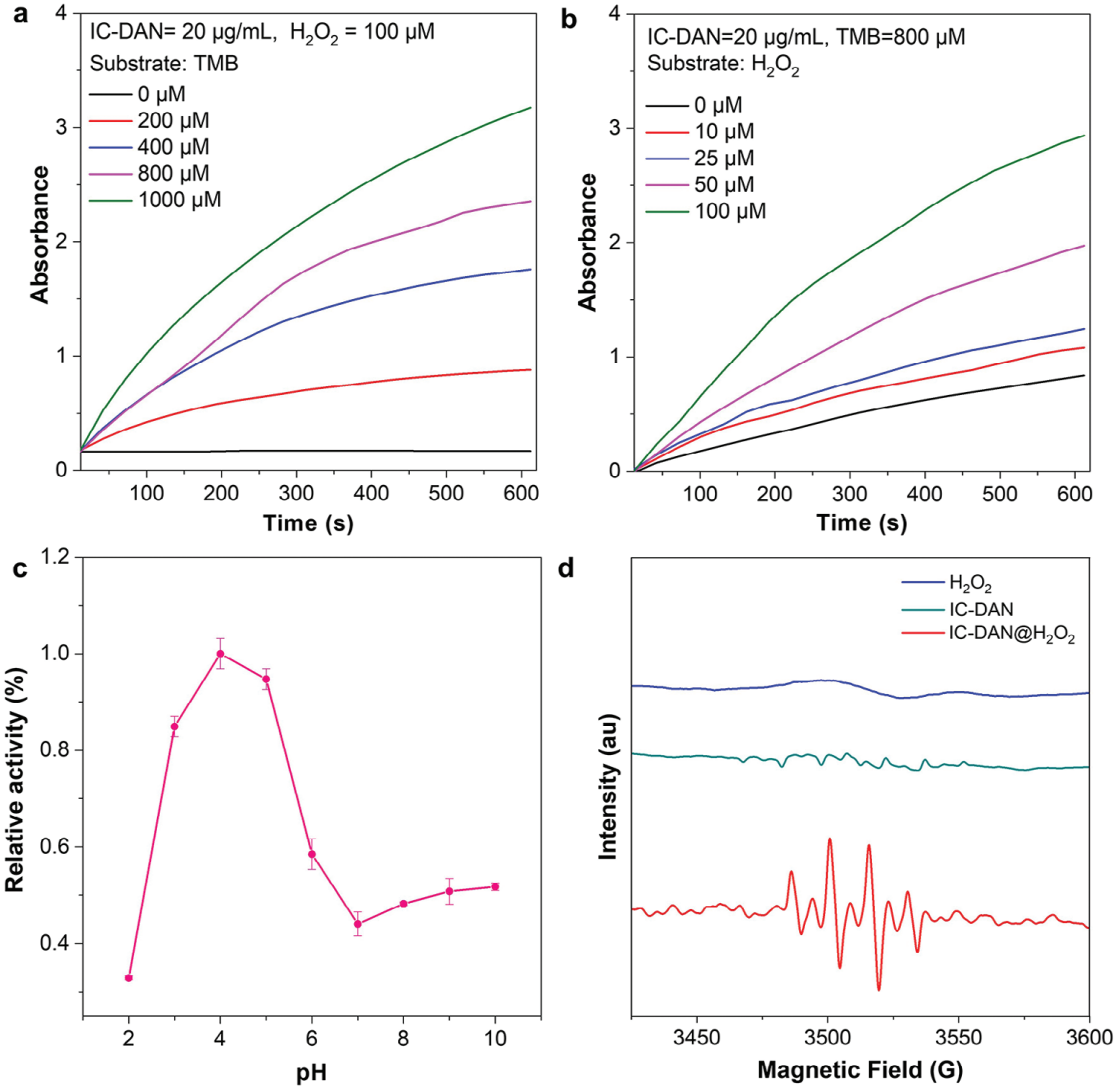
Steady‐state kinetic assay of IC‐DAN for a) TMB and b) H_2_O_2_, respectively. c) Initial velocity profile of TMB chromogenic curves catalyzed by IC‐DAN (20 µg mL^−1^) and H_2_O_2_ (100 µm) under different pH buffer conditions. d) ESR spectra demonstrating ∙OH generation by H_2_O_2_, IC‐DAN, and IC‐DAN + H_2_O_2._

Peroxidase is an enzyme that can exhibit the capability to catalyze the conversion of hydrogen peroxide (H_2_O_2_) into hydroxyl radicals (∙OH). We thus prepared a series of experiments to test the peroxidase‐like activity of IC‐DAN by 3,3′,5,5′‐Tetramethylbenzidine (TMB) assay and simply altering the reaction conditions including pH, temperature, and the concentrations of IC‐DAN and substrate H_2_O_2_ as well as probe TMB (**Figure** **4**a,b). According to the experimental results, IC‐DAN could generate the cytotoxic substance ∙OH through incubation with H_2_O_2_ and further oxidize colorless TMB into blue TMB (oxTMB). As infected wound tissues typically exhibit a higher acidity compared to normal tissues, the enzymatic activity of IC‐DAN was measured at various pH levels to assess its catalytic performance in the context of infected wound environments. As presented in Figure 4c, the result indicated that the catalyst action of IC‐DAN under weak acidic conditions (pH 4) was significantly enhanced than that under neutral conditions. When the reaction temperature was changed, the findings revealed that the POD‐like activity of IC‐DAN was optimum at 40 °C (Figure S11, Supporting Information).

As depicted in Figure 4a,b, the production of oxTMB could be detected through the measurement of absorbance at 652 nm with the increased concentrations of the IC‐DAN, TMB, and H_2_O_2_ in the appropriate concentration range, indicating the robust POD‐like activity of IC‐DAN. To evaluate the Michaelis constant (*K*_m_), conversion rate (*V*_0_), and the maximal rate of conversion rate (*V*_max_), we employed standard Michaelis–Menten curves by altering the concentrations of TMB and H_2_O_2_. Based on the pertinent experimental results, we calculated *K*_m_ = 2.37 × 10^2^ mm, *V*_max_ = 2.21 × 10^−6^ m s^−1^ (Figure S12, Supporting Information). The values of *K*_m_ and *V*_max_ for SAN toward H_2_O_2_ were calculated as 8 × 10 mm and 1.16 × 10^−6^ m s^−1^, respectively (Figures S12 and S13, Supporting Information). Compared with SAN, IC‐DAN exhibited competitive catalytic activities with *K*_m_ of 0.17 mm and *V*_max_ of 1.53 × 10^−6^ m s^−1^, respectively. The calculated values of *K*_cat_ for IC‐DAN toward H_2_O_2_ and TMB were 4.92 and 6.49 S^−1^, respectively, exhibiting higher values compared with those of SAN (Tables S3 and S4, Supporting Information). These results indicate that IC‐DAN can serve as an efficient dual‐atom peroxidase enzyme.

∙OH is known to promote the oxidation of cellular components, consequently triggering bacterial apoptosis.^[^ ^7^ ^]^ The generation of ∙OH radicals was corroborated through electron spin resonance (ESR) spectroscopy. The experimental results (Figure 4d) showed that the signal intensity from ∙OH increased significantly in IC‐DAN when 100 µm of H_2_O_2_ was added. This result well demonstrated that IC‐DAN could catalyze the conversion of H_2_O_2_ into form ∙OH.^[^ ^14^ ^]^

### 2.4 In Vitro Photothermal Effect and Antibacterial Activity of IC‐DAN

**Figure 5:**
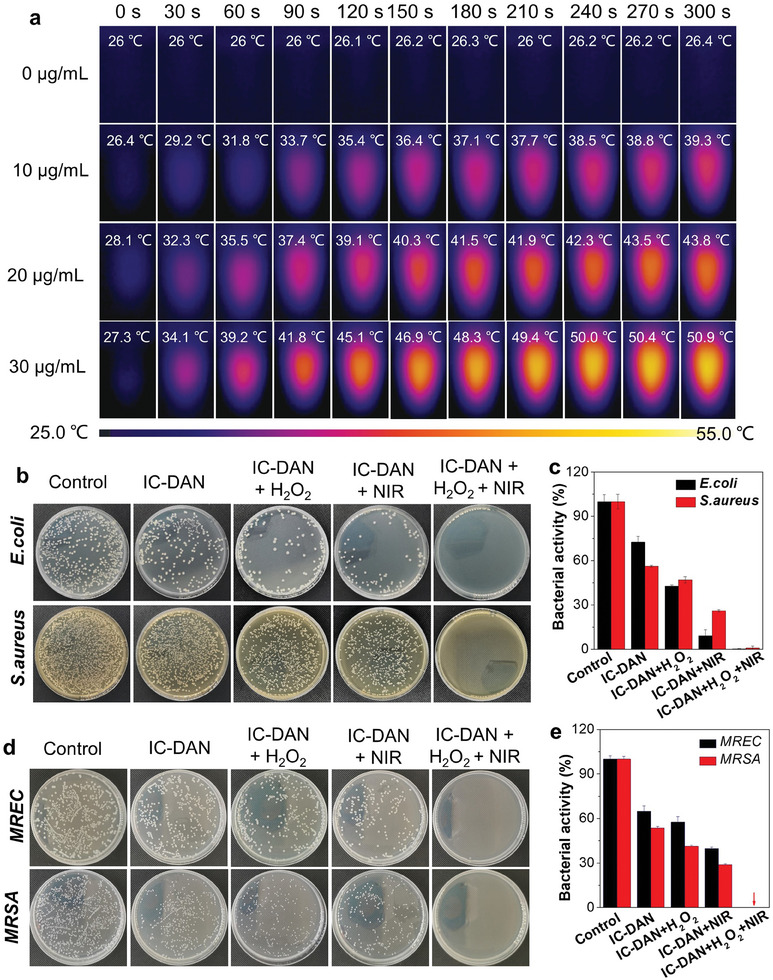
a) IR images and corresponding temperature. b) Digital photographs of remaining bacteria‐inoculated agar plates and c) corresponding bacterial activity after treatment of respective conditions. Upper panel: *E. coli* cells; lower panel: *S. aureus* cells. Values are presented as mean ± SD. d) Digital photographs of remaining bacteria‐inoculated agar plates and e) corresponding bacterial activity after treatment of respective conditions. Upper panel: *E. coli* cells; lower panel: *S. aureus* cells. Values are presented as mean ± SD.

It has been well‐documented that some nanozymes possess superior photothermal properties.^[^ ^32^ ^]^ So we attempted to check whether IC‐DAN also had photothermal conversion ability. The 808 nm near‐infrared laser was used to investigate the photothermal effect of IC‐DAN. Temperature changes of IC‐DAN at different concentrations and exposed to different power densities were evaluated by infrared imaging. According to the results in **Figure** **5**a, the temperatures of the solution rapidly increased to 43.8 °C when IC‐DAN was at a concentration of 20 µg mL^−1^ and the irradiance of the 808 nm near‐infrared (NIR) laser was quantified at 0.8 W cm^−2^. Therefore, IC‐DAN exhibited superior photothermal conversion ability.

**Figure 6:**
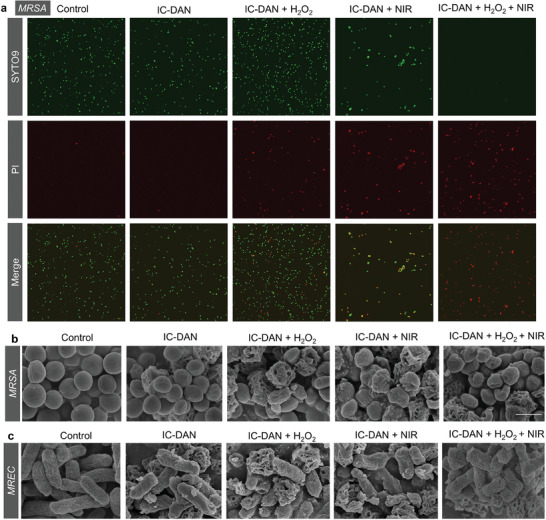
a) Confocal images of Calcein‐AM and PI staining MRSA bacterial cells. b,c) SEM images of MRSA and MREC bacterial cells upon varied treatment conditions.

Given the above photothermal conversion ability and peroxidase (POD)‐like activity, it was proposed to hold the potential of photothermal therapy (PTT) and POD‐like mediated combinable antibacterial effects. So the antibacterial ability of IC‐DAN was further assessed in vitro (Figure 5b–e). The antibacterial efficacy of IC‐DAN was evaluated using MRSA (Methicillin‐resistant *S. aureus*) and MREC (Multiresistant *E. coli*) as model organisms.^[^ ^33^, ^34^ ^]^ After different treatments, the bacterial suspensions were grown on LB agar by a series of dilutions for 16 h. In the group treated with IC‐DAN alone, the number of bacteria colonies indicated that the remaining bacterial activities were 64.92% and 53.67% for MREC and MRSA, respectively. We presumed that the antibacterial effect was due to the peroxidase catalytic effect of IC‐DAN. To verify this point, H_2_O_2_ (100 µm) was added to the bacteria suspension, and we indeed observed that the remaining bacterial activities declined to 57.53% and 41.28% for MREC and MRSA, respectively. In the NIR irradiated groups (IC‐DAN + NIR), the temperatures of the bacteria suspensions increased quickly within 5 min of laser irradiation and the remaining bacterial activities were 39.81% and 28.84% for MREC and MRSA, respectively, indicating a certain PTT ability of IC‐DAN. Impressively, after the combined treatment from IC‐DAN, H_2_O_2,_ and NIR irradiation, at elevated populations (10^8^ CFU mL^−1^), it demonstrated complete eradication of the bacterial cells. These results indicated that IC‐DAN exhibited excellent antibacterial capability when it played two roles in peroxidase‐like and hyperthermia activities at the same time. Furthermore, we evaluated the antibacterial activities of IC‐DAN by fluorescence microscope. A live/dead dual‐color fluorescent stain kit was employed in this experiment to differentiate the antibacterial activities in vitro, where SYTO9 can stain all cells and displays green fluorescence under excitation at 488 nm, while propidium iodide (PI) can only penetrate dead cells and manifests red fluorescence under excitation at 561 nm. As illustrated in **Figures** **6**a and S14 (Supporting Information), most of the bacteria exhibit green fluorescence when treated with IC‐DAN alone. For the group of “IC‐DAN + H_2_O_2_” or “IC‐DAN + NIR”, a small proportion of bacteria exhibit red fluorescence, which was ascribed to the single peroxidase‐like activity or photothermal effect of IC‐DAN. In contrast, strong red fluorescence could be detected in the group of “IC‐DAN + H_2_O_2_ + NIR”, indicating that the bacteria was substantially damaged by the synergetic therapy from peroxidase‐like functionality and its hyperthermia‐inducing properties of IC‐DAN. These results were well consistent with those of the in vitro antibacterial experiments. Based on these in vitro data, it could be concluded that IC‐DAN possesses outstanding antibacterial capability.

To further explore the antibacterial mechanism of IC‐DAN, we examined the structural integrity of bacterial membranes for MREC and MRSA through SEM (Figure 6b,c).^[^ ^35^ ^]^ In the control group, we observed the complete rod‐like structures of MREC bacteria and the intact spherical structures of MRSA bacteria. After treatment with IC‐DAN, the integrity of bacterial cell membranes showed slight damage, indicating that the treatment with IC‐DAN nanomaterials alone was not enough to cause widespread disruption to the integrity of bacteria membranes. However, after treatment with IC‐DAN under H_2_O_2_ and NIR laser irradiation, the cell membranes of MREC and MRSA were severely damaged, accompanied by significant shrinkage, visible collapses, and cracks at the cell membrane surfaces. As a result, we can conclude that IC‐DAN generates quick sterilization by damaging the bacterial membranes via the photothermal action mediated by NIR and POD‐like activity boosted by H_2_O_2_.

### 2.5 In Vivo Eradication of Wound Infections

**Figure 7:**
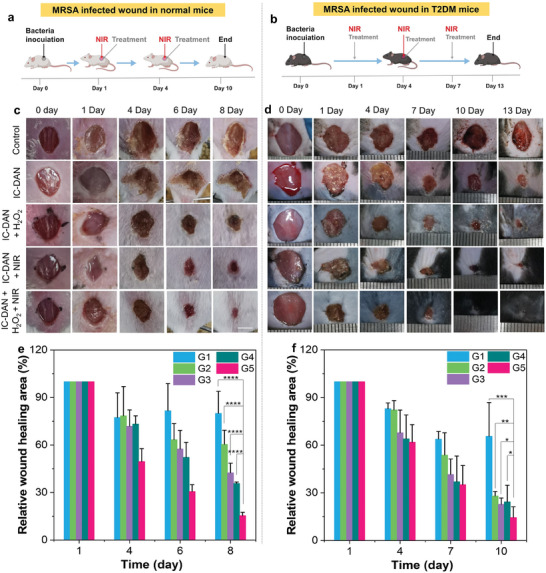
a) Schematic overview of the IC‐DAN antimicrobial treatment regimen in mice models infected with MRSA (all animal experiments were authorized by the Institutional Animal Care and Use Committee of Southern Medical University (IACUC Number: SMUL2022216)). b) Diagrammatic illustration of the therapeutic regimen for combating infection in mice with type 2 diabetes infected by MRSA. c) Accompanying digital images depicting the condition of mice afflicted with MRSA infection. Scale bar: 5 mm. d) Accompanying digital images illustrating the condition of mice with type 2 diabetes following MRSA infection. e) Relative wound healing area in MRSA‐infected mice (n = 5). Values are presented as mean ± SD. f) Relative wound healing area in MRSA‐infected type 2 diabetes mice (n = 5). G1, control; G2, IC‐DAN; G3, IC‐DAN + H_2_O_2_; G4, IC‐DAN + NIR; and G5, IC‐DAN + H_2_O_2_ + NIR. ^****^ *p* <0.0001, ^***^ *p* <0.001, ^**^ *p*<0.01, ^*^ *p* <0.05.

In light of the excellent antibacterial ability of IC‐DAN in vitro, we further investigated the therapeutic effects of IC‐DAN on infected wounds in both normal mice and type 2 diabetic mice (**Figure** **7**a,b). After the successful construction of infected diabetic mice, the cohort of mice was subject to a randomized allocation into five distinct groups: G1, control; G2, IC‐DAN; G3, IC‐DAN + H_2_O_2_; G4, IC‐DAN + NIR; and G5, IC‐DAN + H_2_O_2_ + NIR. To investigate the healing efficacy of infected wounds, the areas of bacterial infection sites were monitored every three days. Taking the model of type 2 diabetic mice (Figure 7d) as the example, it was worth noting that the mice in G5 (IC‐DAN + H_2_O_2_ + NIR) achieved nearly full healing on day 13, but only parts of the wound were healed for the other groups and obvious wound scars remained in the control group. After statistical analysis, the relative wound unhealed area in G5 reached 14.51% of the initial size, while G4 reached 24.36%, G3 reached 22.82%, G2 reached 28.07%, and G1 reached 65.62%, respectively (Figure 7f). Additionally, the related results demonstrated the wound healing speed in G5 was significantly faster than that of the other groups, especially compared with the mice in G1. Regarding the normal mice infected by MRSA (Figure 7c–e) and MREC (Figure S22a,b, Supporting Information), the wound in G5 also exhibited the fastest healing. Meanwhile, the mice became mentally better and gained more weight than the other groups after treated with (IC‐DAN + H_2_O_2_ + NIR) (Figures S15a and S22c, Supporting Information), and the healing time was shorter compared with that of the type 2 diabetic mice. After comparing the healing process of infected wounds in ordinary mice and diabetic mice, it is evident that the wound healing cycle of diabetic mice is longer (13 days), and the weight of the mice is lower (Figure S15b, Supporting Information). This phenomenon is also consistent with the fact that the wound healing rate of diabetic patients is much lower than that of non‐diabetic patients. Meanwhile, the investigation into the wound healing mechanism involved the application of hematoxylin and eosin (H&E) staining, as well as Masson's trichrome staining techniques. Previous research has shown that wound healing is a multi‐step biological process, including hemostasis, inflammation, migration, proliferation, reepithelialization, and maturation.^[^ ^7^, ^9^ ^]^ As shown in Figures S16–S23 (Supporting Information), we could observe an obvious boundary between normal tissue and the wound in G1 and G2 on day 13. The wound portion was filled with a considerable number of inflammatory cells as revealed by H&E staining of wound tissue and most collagen fibers were lost according to Masson's trichrome staining. The borders between normal tissue and the wound were becoming blurred after treated with IC‐DAN + H_2_O_2_ or IC‐DAN under NIR irradiation. In comparison, the wound portion was gradually replaced by normal tissue in G5. There were significantly fewer inflammatory cells in G5 than in the other groups and lots of blood vessels and collagen fibers were formed in G5. Thus, the findings of animal experiments solidly indicate that IC‐DAN has outstanding wound healing capabilities with photothermal effect and POD‐like activity.

We all know that biocompatibility and biosafety are very vital for the biological application of nanomaterials. Thus, normal mammalian cells including human dermal fibroblasts (HDF) cells and human umbilical vein endothelial cells (HUVEC)  were used to assess the cytocompatibility of IC‐DAN in vitro. The results of CCK8 assay indicated that the cell viabilities of both HDF cells and HUVECs were still above 80% at working concentrations (20 µg mL^−1^) of IC‐DAN nanomaterials after co‐incubated for 24 h (Figure S18a, Supporting Information). Moreover, we employed a hemolysis experiment to evaluate the biocompatibility of IC‐DAN using water‐treated and PBS‐treated groups as positive and negative controls. As shown in Figure S18b (Supporting Information), there was no evident hemolysis when incubated with IC‐DAN at working concentrations, indicating that IC‐DAN was highly biocompatible. On day 13, the major organs comprising the heart, liver, spleen, lung, and kidney were surgically excised after anesthesia and H&E staining to explore the potential systemic toxicity of IC‐DAN. As illustrated in Figure S19 (Supporting Information), there were no significant changes in organ tissue H&E stained. Furthermore, hematological analysis revealed no obvious changes, including blood cell count (RBC), mean corpuscular hemoglobin (MCH), mean corpuscular hemoglobin concentration (MCHC), hemoglobin (HGB), mean corpuscular volume (MCV), and platelet count (PLT) among different treatment therapy (Figures S20 and S21, Supporting Information). We also discovered no obvious differences in hepatic and renal toxicity using blood biomarkers such as blood urea nitrogen (BUN), alanine transaminase (ALT), aspartate transaminase (AST), and creatinine. All these data confirm that IC‐DAN holds good biocompatibility and biosafety. Considering the collective antibacterial results, it can be inferred that IC‐DAN holds significant potential for application as a clinical antibacterial agent.

## 3 Conclusion

In summary, we constructed a new iron coupling dual‐atom peroxidase nanozyme (IC‐DAN) containing FeN_4_–FeN_4_ active center structure. The exceptional efficiency of peroxidase‐mediated hydroxyl radical production under conditions simulating physiological levels of H_2_O_2_ has been comprehensively substantiated through ESR spectra and conventional enzymatic kinetics. Furthermore, DFT calculations and XAS analysis elucidate the favorable role of the coupled Fe system in facilitating the generation of toxic ∙OH radicals. The exceptional efficiency of peroxidase‐induced hydroxyl radical production has undergone rigorous validation through ESR spectra and conventional enzymatic kinetics assessment under conditions reflecting physiologically relevant levels of H_2_O_2_. Additionally, DFT and XAS analysis elucidates that the coupling diatomic system provides a favorable environment for the generation of toxic ∙OH radicals. Additionally, combining with the efficient photothermal conversion ability, IC‐DAN achieved effective antibacterial therapy and significantly prolonged the survival of type 2 diabetic mice. Taken together, our results and findings of the work present a notable breakthrough in the advancement of high‐performance artificial enzymes. This offers valuable insights for the prospective design and evolution of enzymatic materials, ensuring exceptional antibacterial performance and confirmed biocompatibility.

## Conflict of Interest

The authors declare no conflict of interest.

## Supporting information

Supporting Information

## Data Availability

The data that support the findings of this study are available from the corresponding author upon reasonable request.
