# Supplementary material for: Construction of Interlayer Coupling Diatomic Nanozyme with Peroxidase‐Like and Photothermal Activities for Efficient Synergistic Antibacteria
Source: Adv Sci (Weinh). 2024 Mar 9;11(20):2305823. doi: 10.1002/advs.202305823 (PMC11132033; doi:10.1002/advs.202305823)
Supplement: Supplementary file 1 — Supporting Information [file ADVS-11-2305823-s001.pdf]

ADVANCED SCIENCE
Open Access

Supporting Information

for *Adv. Sci.*, DOI 10.1002/advs.202305823

Construction of Interlayer Coupling Diatomic Nanozyme with Peroxidase-Like and Photothermal Activities for Efficient Synergistic Antibacteria

Xiudong Shi, Jie Lv, Shuangling Deng, Fang Zhou, Jiangang Mei, Lei Zheng and Jing Zhang**

Supporting Information

Construction of Interlayer Coupling Diatomic Nanozyme with Peroxidase-Like and Photothermal Activities for Efficient Synergistic Antibacteria

Xiudong Shi, Jie Lv, Shuangling Deng, Fang Zhou, Jiangang Mei, Lei Zheng, and Jing Zhang**

Department of Laboratory Medicine
Nanfang Hospital, Southern Medical University
Guangzhou, 510515, China
Email: nfyyzhenglei@smu.edu.cn; zjsilence@i.smu.edu.cn

1. Experimental Section

Preparation of ZIF-8 nanocubes. The ZIF-8 nanocubes were synthesized using CTAB as a capping agent by a chemcial bath depostion (CBD) method. First of all, 0.36 g $ZnNO_3 \cdot 6H_2O$ was dissolved in 100 mL deionized water. 5.60 g Hmim and 0.01 g CTAB were dissolved in 70 mL DI water. The above mixture was strongly stirred for 3 h at room temperature to form homogeneous solution. Then, the above solution was centrifugated, washed with methanol and dried overnight at 70 °C to obtain ZIF-8 nanocrystals.

Preparation of SAN: In a typical procedure, 0.40 g ZIF-8 and 0.280 g cyclopentadienyliron dicarbonyl dimer were individually placed in a quartz boat. The boat was heated at 350 °C for 2 h with a heating rate of 3 °C min^{-1} under an Ar atmosphere. Afterthat, the above sample was further heated at 950 °C for 2 h with a heating rate of 4 °C min^{-1}. After cooling down to room temperature, the obtained powder was leached with 0.5 M H_2SO_4 at 80 °C for 12 h, followed by washing with DI water and annealing at 950 °C to form the final SAN.

Preparation of Coupling Diatomic Nanozyme. In a typical procedure, 0.40 g ZIF-8 and 0.40 g cyclopentadienyliron dicarbonyl dimer were individually placed in a porcelain boat. The boat was heated at 350 °C for 2 h with a heating rate of 3 °C min^{-1} under an Ar atmosphere. Afterthat, the above sample was further heated at 950 °C for 2 h with a heating rate of 4 °C min^{-1}. After cooling down to room temperature, the obtained powder was leached with 0.5 M H_2SO_4 at 80 °C for 12 h, followed by washing with DI water and annealing at 950 °C to form the final IC-DAN .

2. Characterization of Materials

The microstructure of the as-prepared samples were monitored by using a JEM-2100 (JEOL) TEM at 100 kV and a JEM-2100F HRTEM at 200 kV. High-angle annular dark-field scanning transmission electron microscopy (HAADF-STEM) was performed on a Titan 80-300 scanning/transmission electron microscope operated at 300 kV, equipped with a probe spherical aberration corrector. The Powder X-ray diffraction (XRD) patterns were recorded on a TD-3500 powder dilatometer (Tongda, China) operating at 30 kV and 20 mA with Cu Kα radiation sources. The X-ray photoelectron spectrum (XPS) was carried out on ESCALAB 250 (Thermo-Fisher Scientific, USA). The Raman spectra measurements were performed by a Raman spectroscopy (inVia,

Renishaw, China) with a 633 nm laser source. The Fe K-edge X-ray absorption near-edge structure (XANES) and Extended X-ray Absorption Fine Structure (EXAFS) data were collected on the 06ID-1 Hard X-ray Micro Analysis (HXMA) beamline at the Canadian Light Source.

$A = kbc$

$V_0 = \triangle A/kb\triangle t$

$V_0 = V_{\max} \bullet [S]/(K_m + [S])$

Where A represents the absorbance values, k represents the molar extinction coefficient. In this case, k = 39000 M cm^{-1}. c refers to the concentration of the chromogenic substances while t refers to time.

In this equation, V_0 is the conversion rate, V_{max} is the maximal rate of conversion rate, K_m is the Michaelis constant, and [S] is the substrate concentration. The K_m is equal to the substrate concentration at which the conversion rate is half of V_{max}. K_m means the affinity of the enzyme for the substrate namely a lesser K_m value implies a better affinity.

3. Calculation Methods

The spin-polarized density functional theories (DFT) were carried out by using the Vienna Ab initio Simulation Package (VASP)[1]. The Perdew-Burke-Ernzerhof generalized-gradient approximation functional was used to describe the interaction between electrons [2]. The energy cutoff was set to 400 eV. The 2×2×1 Gamma-Centered k-points grid was set during the calculations. The vacuum region was set to be 15 Å in z direction to prevent the interaction between two adjacent surfaces. The energy convergence was set to 10^{-5} eV. The convergence criteria of the forces were selected as 0.02 eV $Å^{-1}$.

The reaction Gibbs free energy (ΔG) is defined as $\Delta G = \Delta E + \Delta E_{ZPE}$ - TΔS, ΔE is the reaction energy, ΔE_{ZPE} is zero-point energies, T is the temperature (298.15 K), ΔS is the difference in entropy from vibrational frequency calculations. The entropies of gas phase H_2O_2, H_2 and H_2O are obtained from the NIST database with standard condition [3].

4. Biological Experiments

Instruments. Fluorescence imaging by using an inverted confocal fluorescence microscope (FV3000, Olympus). 808 nm near-infrared (NIR) laser was bought from Jiuyi Laser Technology (Beijing, China). The thermal imaging of IC-DAN solution and mice was imaged by an infrared thermal camera (HIKVISION, China). Absorbance was measured by a microplate reader (Cytation5, BioTek).

Materials. 3,5,3',5'- tetramethylbenzidine (TMB) were purchased from Sigma-Aldrich. H_2O_2 solutions were purchased from Macklin (Shanghai, China). LIVE/DEAD BacLight Bacterial Viability Kit was purchased from Thermo Fisher Scientific. Human Umbilical Vein Endothelial Cells (HUVECs) and human diploid fibroblasts (HDFs) cells were purchased from the American Type Culture Collection (ATCC). Phosphate buffered saline (PBS), high glucose Dulbecco modified eagle medium (DMEM), streptomycin/penicillin, and fetal bovine serum (FBS) were obtained from Gibco. Lysogenic broth (LB) mediums and LB agar were purchased from Solarbio (Beijing, China). streptozotocin (STZ) was purchased from Sigma-Aldrich. High-fat feed was purchased from Changsheng biotechnology (Liaoning, China). Gram-positive Staphylococcus aureus (SA, ATCC29213), methicillin-resistant Staphylococcus aureus (MRSA), Gram-negative Escherichia coli (EC, ATCC25922,), and multidrug-resistant Escherichia coli (MREC) were collected from the clinic.

Measurement of Peroxidase-Like Activity. The peroxidase (POD)-like activity of IC-DAN was measured using TMB as a probe in the HAc-NaAc buffer. The absorbance at 652 nm was recorded using a Cytation 5 microplate reader. To study the effect of pH on POD-like activity of IC-DAN, we added IC-DAN (20 μg mL^{-1}) and 800 μM TMB solution into HAc-NaAc buffer (pH 2.0, 3.0, 4.0, 5.0, 6.0, 7.0, 8.0, 9.0 and 10.0), respectively. The mixture was incubated for 10 min and the final 652 nm absorbance of the mixture was detected by a microplate reader. To further measured the kinetic assays of IC-DAN, the kinetic assays of IC-DAN (20 μg mL^{-1}) with TMB as the substrate were performed by adding 100 μM H_2O_2 and different concentrations of TMB solution (0, 200, 400, 800, 1000 μM) in a 0.01 M HAc-NaAc buffer (pH = 4). The kinetic assays of IC-DAN (20 μg mL^{-1}) with H_2O_2 as the substrate were performed by adding 800 μM TMB and different concentrations of H_2O_2 solution (0, 10, 25, 50, 100 μM) in a HAc-NaAc buffer (pH = 4). To measure the effect of temperature on the POD-

like activity of IC-DAN. The kinetic assay was conducted at different temperatures (20, 30, 40, 50, and 60°C) in a 0.01 M HAc-NaAc buffer (pH = 4) with IC-DAN (10 μg mL^{-1}) and 800 μM TMB and 100 μM H_2O_2. The absorbance of the solution was monitored at different reaction times for 10 min. By fitting the absorbance data to the Michaelis-Menten equation, we could calculate the catalytic parameters, which present the connection between the substrate and IC-DAN.

ESR experiments for hydroxyl radical determination. During the ESR measurements, BMPO was used for hydroxyl radicals scavengers, trapping the radicals and forming adducts that are detectable by ESR spectroscope. In a typical assay, 20 μL of IC-DAN(1000 μg mL^{-1}) and 50 μL of H_2O_2 (100 μM) were added into 100 μL of PBS buffer solution with different pH conditions (pH = 4.0) containing 40 μM of BMPO. The system was then subjected to vortex for 1 min before being transferred to the quartz tube for ESR measurements. The temperature induced ESR measurement was accomplished by coincubating the system in the waterbath at 45℃ for 1 min prior to the measurement.

Photothermal Effect of IC-DAN. IC-DAN was dispersed in ultra-pure water at three concentrations (10, 20, or 30 μg mL^{-1}). Then 100 μL solution was added to a centrifuge tube and irradiated with an 808 nm NIR laser source at different power densities (0.6, 0.8, or 1.0 W cm^{-2}) for 5 min. The temperatures and photographs of the NIR irradiated IC-DAN solution were recorded at 10 s intervals using an infrared imaging device.

Antibacterial Activity of IC-DAN. Bacterial culture and antibacterial activities in vitro To assess the antibacterial effect of IC-DAN, five groups were established. Bacteria without any treatment served as the control group named G1 and the other experimental groups were treated with IC-DAN (20 μg mL^{-1}) alone named G2, hyperthermia system (IC-DAN + NIR (0.8W cm^{-2})) named G4, peroxidase system (IC-DAN + H_2O_2 (100 μM)) named G3, and their combination (IC-DAN + NIR + H_2O_2) named G5. The specific experimental operation is as follows. Firstly, mono-colonies of MREC, and MRSA on the solid Luria-Bertani (LB) agar plates were transferred to liquid LB broth and grown at 37°C for 12 h at a rotation shaker of 160 rpm. Then, bacteria were collected in the logarithmic phase of growth. The concentration of

bacteria was monitored spectrophotometrically by measuring the optical density at 600 nm (OD600). 100 μL of bacteria suspensions (1×10^8 CFU mL^{-1}) were treated with five different treatments in a 96-well plate and incubated at 37 °C for 4 h, respectively. 100 μL of the bacteria suspension was spread onto LB agar plates and then incubated for 15 h at 37 °C after a series of doubling dilutions. The number of colonies formed units (CFUs) were calculated and imaged the next day. The average CFUs numbers were obtained from three duplicate experiments (n = 3). The formula for calculating the antibacterial ratio is C = (A-B)/A × 100%. Where C indicates antibacterial ratio; A is the average CFUs of the control group, and B is the average CFUs of the experimental group. At the same time, according to the staining kit operating instructions, both MREC and MRSA bacteria were stained with SYTO9 and PI for 15 min in the dark after the same treatment. The SYTO9 could stain with all bacteria and PI only enter through damaged bacteria membranes. Thus, it was employed to label all bacteria with green fluorescence and dead bacteria with red fluorescence. The live and dead bacterial cells were visualized with a fluorescence microscope.

Morphology Observation of Bacteria by SEM Images. After the antibacterial treatment, five groups of the bacterial suspensions were gently washed and fixed with 2 wt% glutaraldehyde overnight at 4 °C. After that, the bacteria were dehydrated by sequential treatments with ethanol for 10 min (30, 50, 70, 90, and 100 %), respectively. Then, bacteria were dried and sprayed with gold on the surface for further field-emission SEM images.

Biosafety Assessment of IC-DAN.

Evaluation of Cytotoxicity *In Vitro.* HDF and HUVEC cells were used for the investigation of the cytocompatibility evaluations. The cells were grown in high glucose DMEM with 1% streptomycin/penicillin and 10% FBS. Cell counting kit-8 (CCK-8) assay was used to measure the cytotoxicity in vitro. The cells were cultured in a 96-well plate (5×10^3 cells per well, five wells for each concentration) at a 37 °C humidified incubator with 5% CO_2 for 12 h. IC-DAN in different concentrations (0,

25, 50, 100, 150, 200 μg mL^{-1}) were added into each well. Then, the cells were co-incubated for 24 h and gently washed with PBS three times. According to the manufacturer's protocol, 10 μL CCK-8 diluted into 100 μL DMEM was subsequently added to each well, and the 96-well plate was kept incubated for another 2 h. The absorbance at 450 nm of each well was measured by a microplate reader to evaluate cell viability. Cells treated with PBS were used as control, and the cell viability was calculated as a percentage relative to the control cells.

Red Blood Cells Compatibility Detection. A hemolysis test was used to analyze the hemocompatibility of IC-DAN *in vitro*. Briefly, Whole blood cells were collected from healthy people by a 2 mL EDTA-K2 anticoagulant tube. Then red blood cells were separated from whole blood by centrifugation at 2,000 g for 5 min and washed with sterile PBS (pH = 7.4) three times. 10% red blood cells were resuspended into PBS and incubated with various concentrations (0, 10, 20, 50, 100, 200 μg mL^{-1}) of IC-DAN at room temperature for 2 h. Subsequently, the samples were centrifuged at 2,000 g for 5 min, and the absorbance of the supernatant at 575 nm was measured by a microplate reader. Red blood cells were treated with either PBS or ultra-pure water to be used as a negative (-) or positive control (+), respectively.

Type 2 Diabetic Mice Wound Infection Model *In Vivo.* To assess the antibacterial effects of IC-DAN *in vivo*, the diabetic mice wound infection model was built. All animal experiments were authorized by the Institutional Animal Care and Use Committee of Southern Medical University (IACUC Number: SMUL2022216). Firstly, 8-weeks-old Male C57BL/6J mice were employed to generate the type 2 diabetes model. After an adaptive diet (25 °C, 12 h day/night cycle) for 7 days, a high-fat diet (D12492, 60% calories from fat) for 8 weeks combined with a daily intraperitoneal injection of STZ (40 mg kg^{-1} day^{-1}) for 7 days were used. The fasting blood glucose concentration was measured at more than 11.1 mmol L^{-1} twice, the mice model was determined to be successful and utilized in the following investigations. Then, the type 2 diabetes mice were randomly divided into five groups (five mice per group). A round wound (d = 8

mm) was cut by surgical scissors on the back of each mouse after anesthesia. Subsequently, MRSA was chosen as the model bacterium for investigating the anti-bacterial effect of IC-DAN in vivo. The wounds were then infected by 20 μL of MRSA bacterial suspension (1×10^8 CFU ml^{-1}). After 24 h, IC-DAN (20 μg mL^{-1}) was dropped on the wound area with further treatment with H_2O_2 or NIR in the corresponding groups and PBS solutions in the control group. The wounds of infection were recorded and photographed every three days.

Hematology and Histological analysis. After 13 days, all mice were sacrificed and the wound tissues were harvested from the mice for further analysis after euthanasia. Whole blood was collected from mice for routine blood tests. Plasma was separated to measure the concentrations of alanine aminotransferase (ALT), aspartate aminotransferase (AST) to analyze the liver function, and blood urea nitrogen (BUN), creatinine (CREA) to assess kidney function, respectively. At the same time, the infected skin tissues in different groups were obtained and fixed with 4wt % formaldehyde solution for hematoxylin and eosin (H&E) staining, Masson staining according to standard operating procedure. The major organs (heart, liver, lung, kidney, and spleen) were excised and fixed with 4 wt % formaldehyde solution for H&E staining. Then the samples were viewed under a digital microscope (Nikon, Japan). After the experiment, the mice were disposed of following the normal authorized protocol.

Statistical Analysis. OriginPro8.5 was used in the statistical analysis. Number of samples and probability values were showed in figures. All data were shown as means ± standard deviation (SD). Student's t-test was used for two groups and one-way analysis of variance (ANOVA) was used for statistical analysis between multiple groups. A probability value of $p < 0.05$ was considered statistically significant (****$p < 0.0001$, ***$p < 0.001$, **$p < 0.01$, *$p < 0.05$), and ns indicated no significant difference.

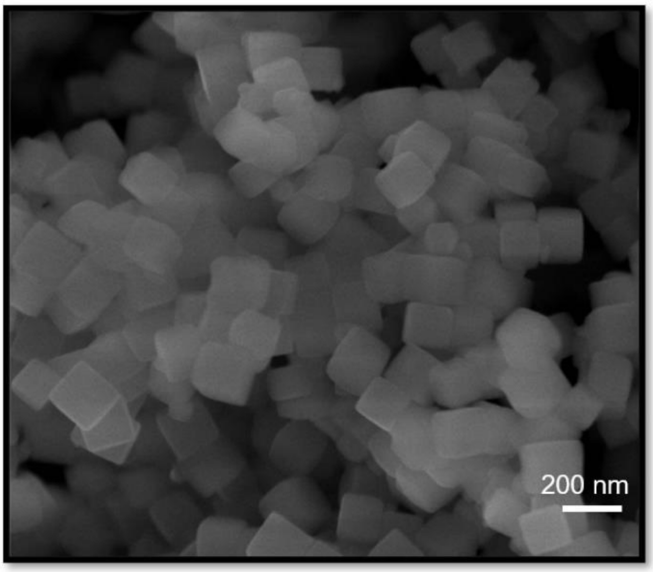

Figure S1. The SEM spectrum of ZIF-8.

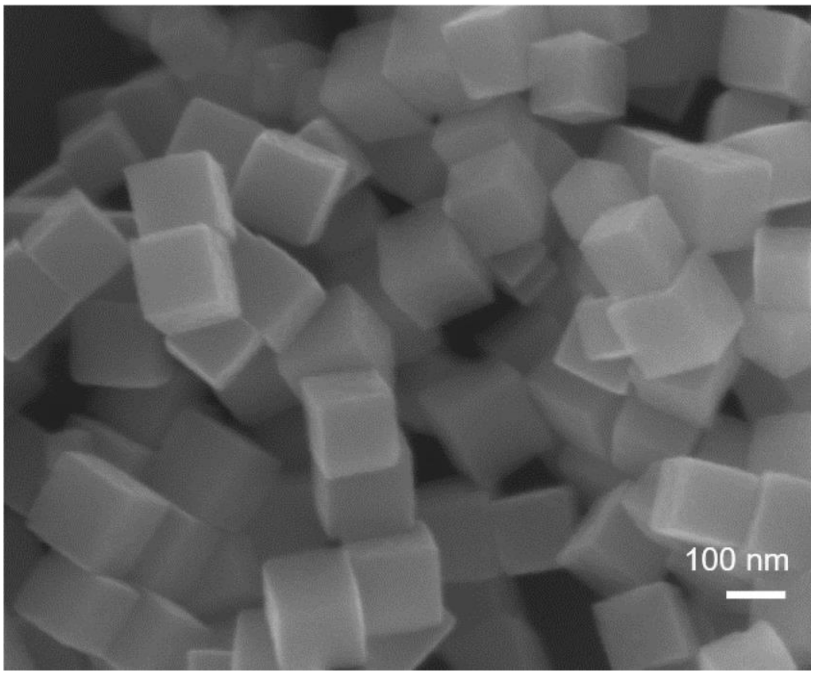

Figure S2. The SEM spectrum of N/CC

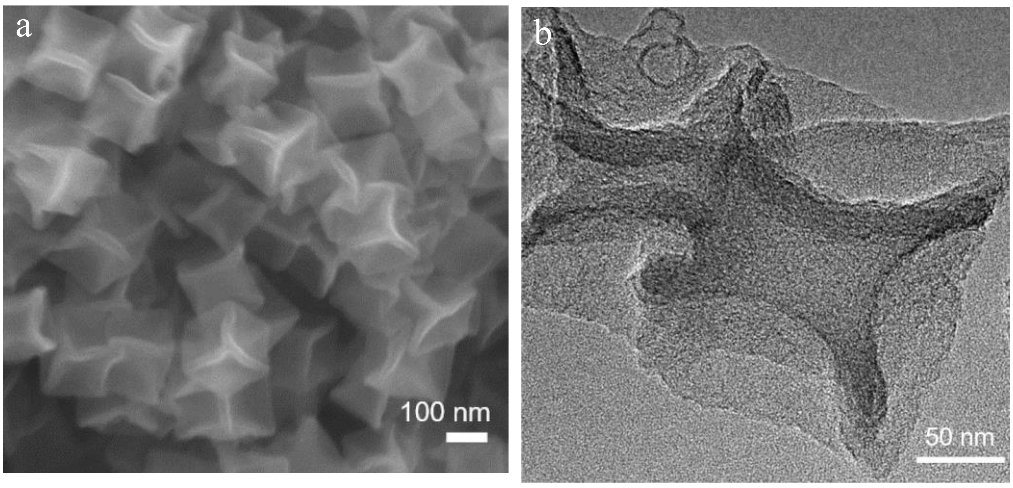

Figure S3. The SEM spectrum (a) and TEM (b) spectrum of SAN

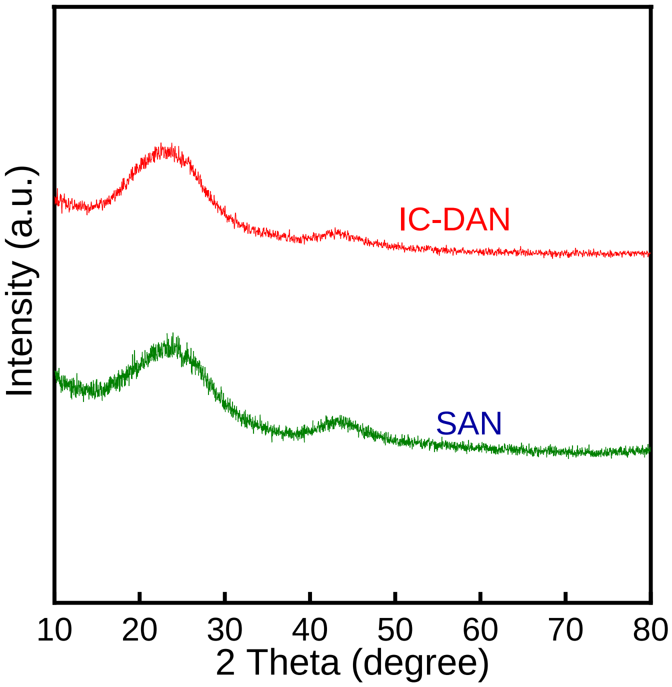

Figure S4. XRD patterns of SAN and IC@DAN

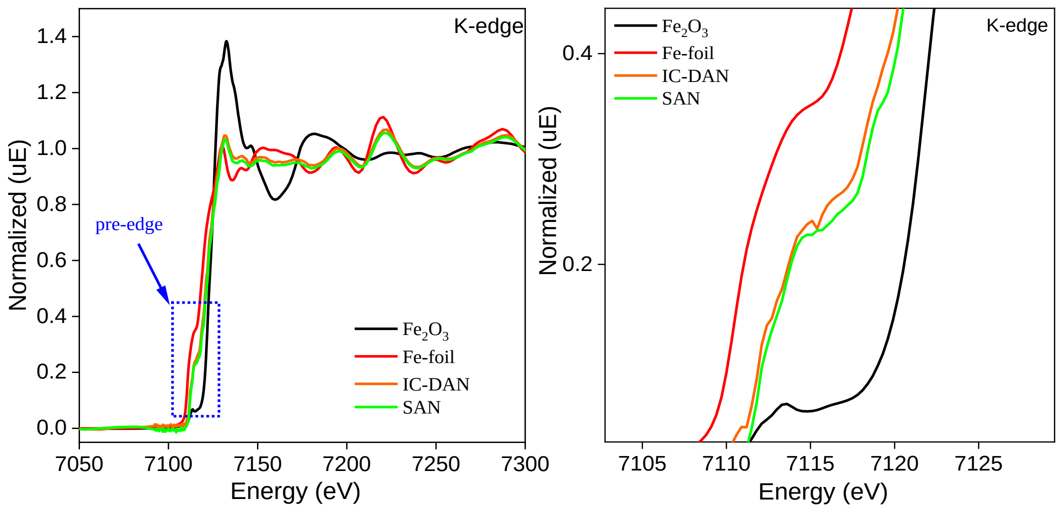

Figure S5. Fe K-edge XANES experimental spectra.

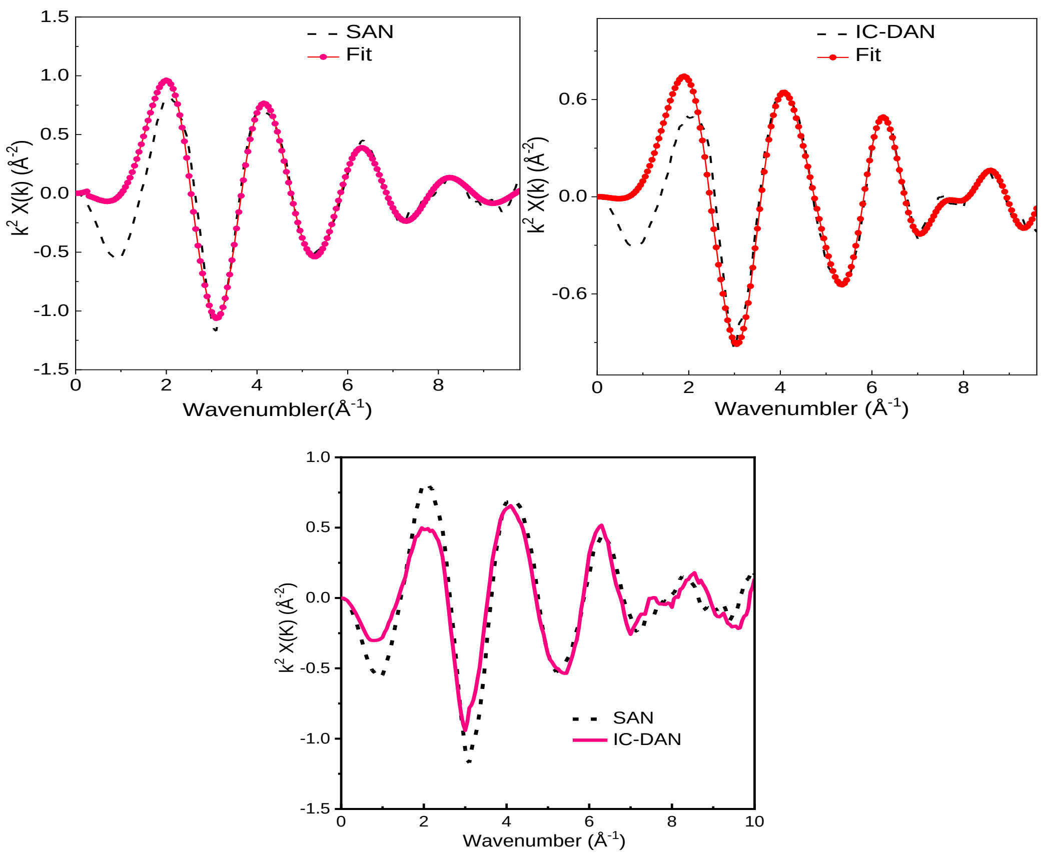

Figure S6. Corresponding EXAFS fitting curves of SAN and IC-DAN at k space

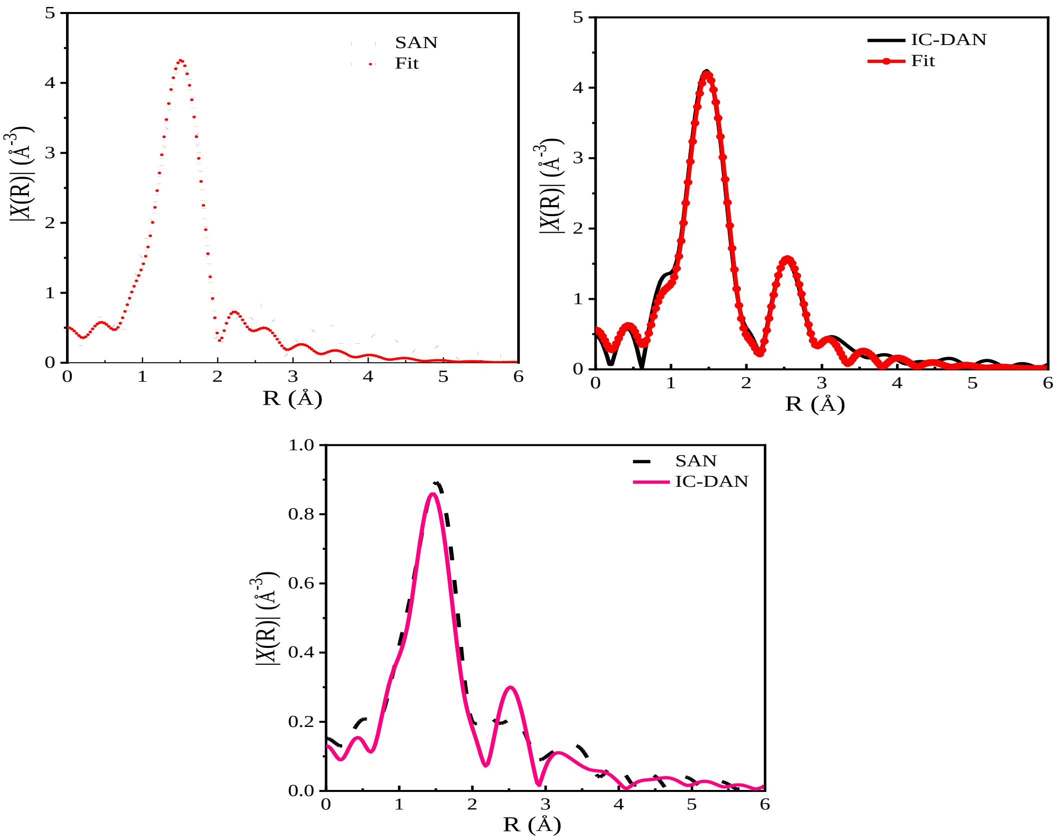

Figure S7. Fe K-edge EXAFS fitting curves of SAN and IC-DAN at R space

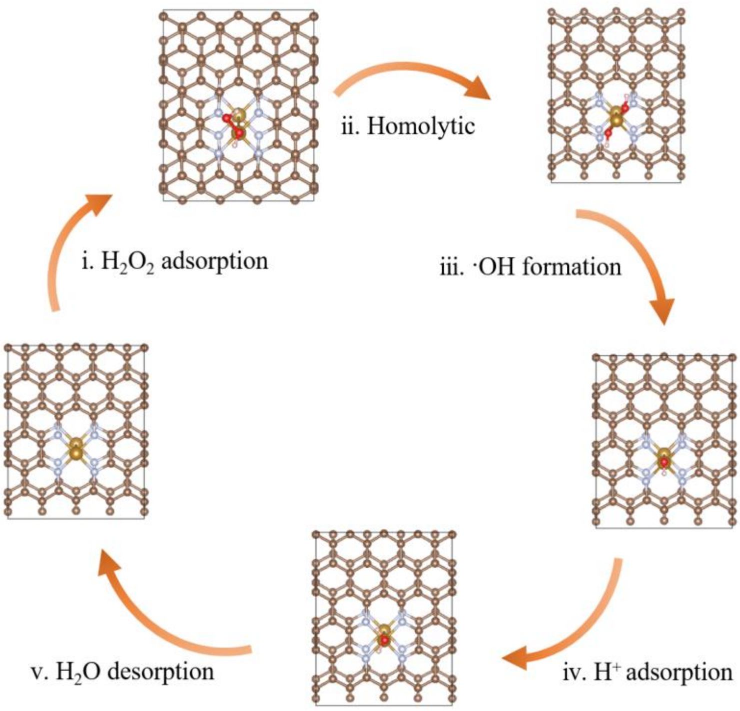

Figure S8. Proposed catalytic mechanism for peroxidase-like reaction on FeN_4-FeN_4.

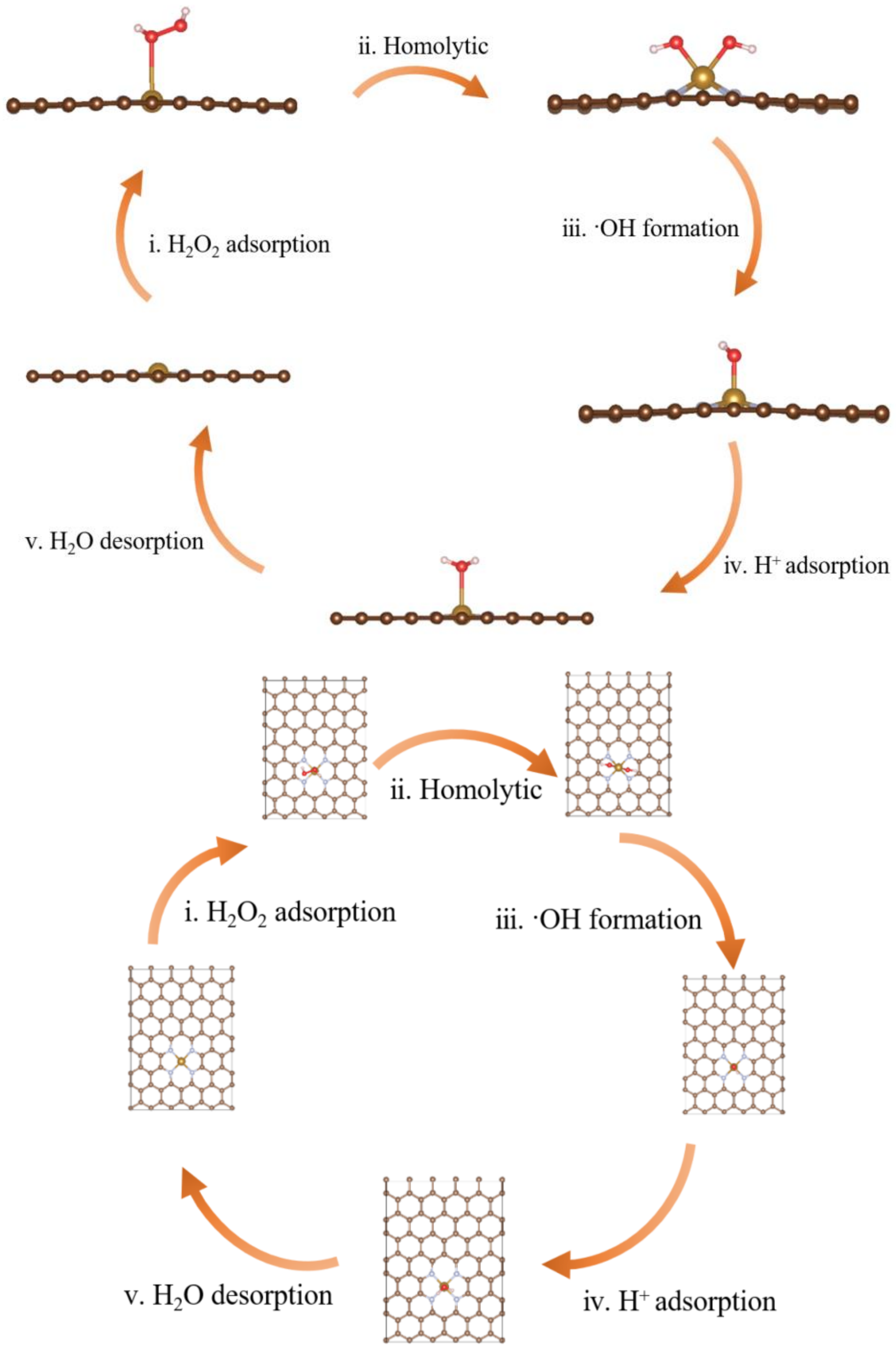

Figure S9. Proposed catalytic mechanism for peroxidase-like reaction on FeN_4 (side view and top view).

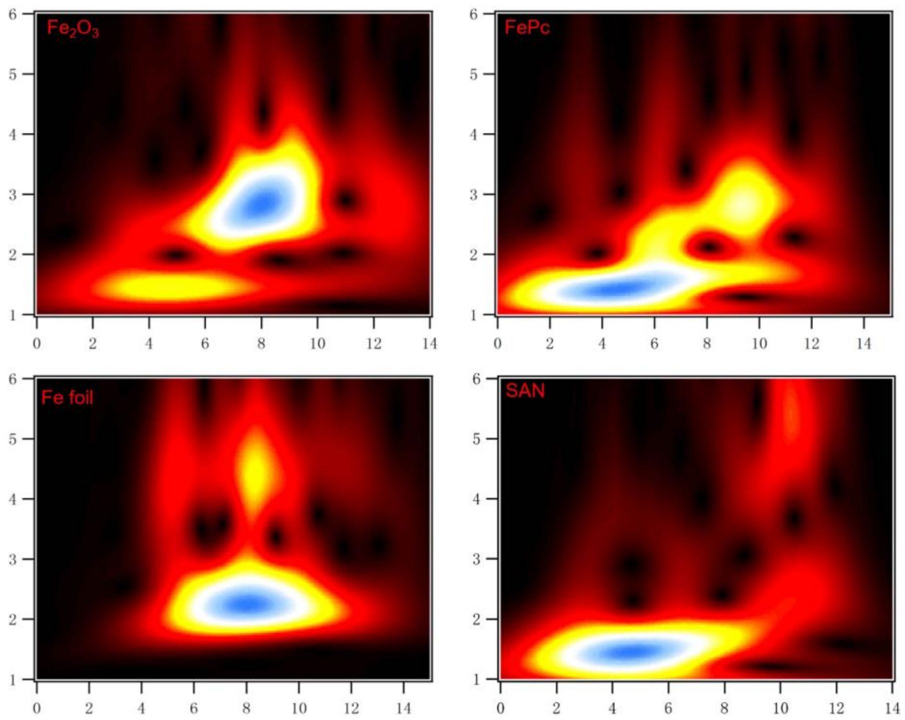

Figure S10. Wavelet transform of the k^3-weighted EXAFS data of Fe for SAN, Fe_2O_3, FePc and Fe foil.

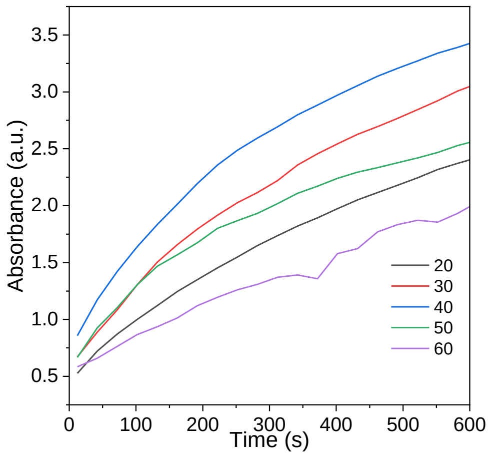

Figure S11. Relative absorbance of TMB chromogenic curves catalyzed by IC-DAN (10 μg mL^{-1}) and H_2O_2 (100 μM) under different temperature conditions.

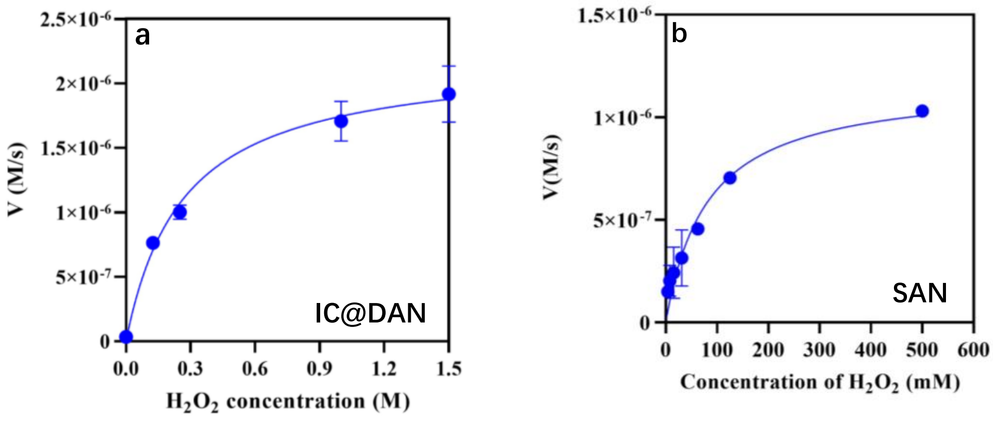

Figure S12. Steady-state kinetic assay of IC-DAN for H_2O_2

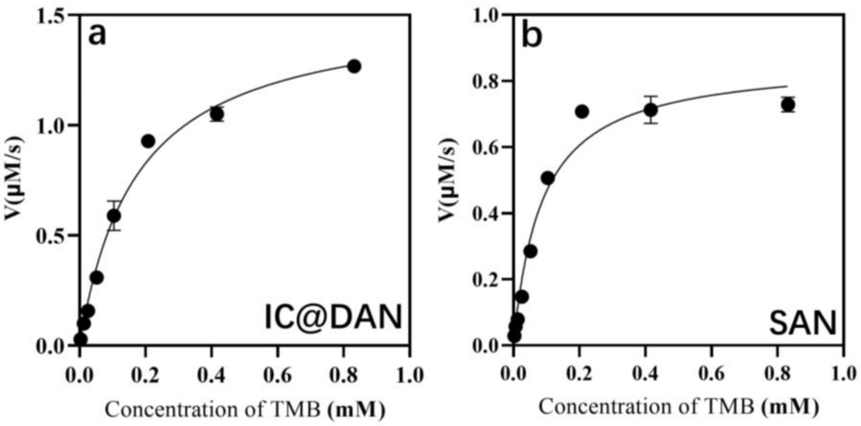

Figure S13. Steady-state kinetic assay of IC-DAN for TMB.

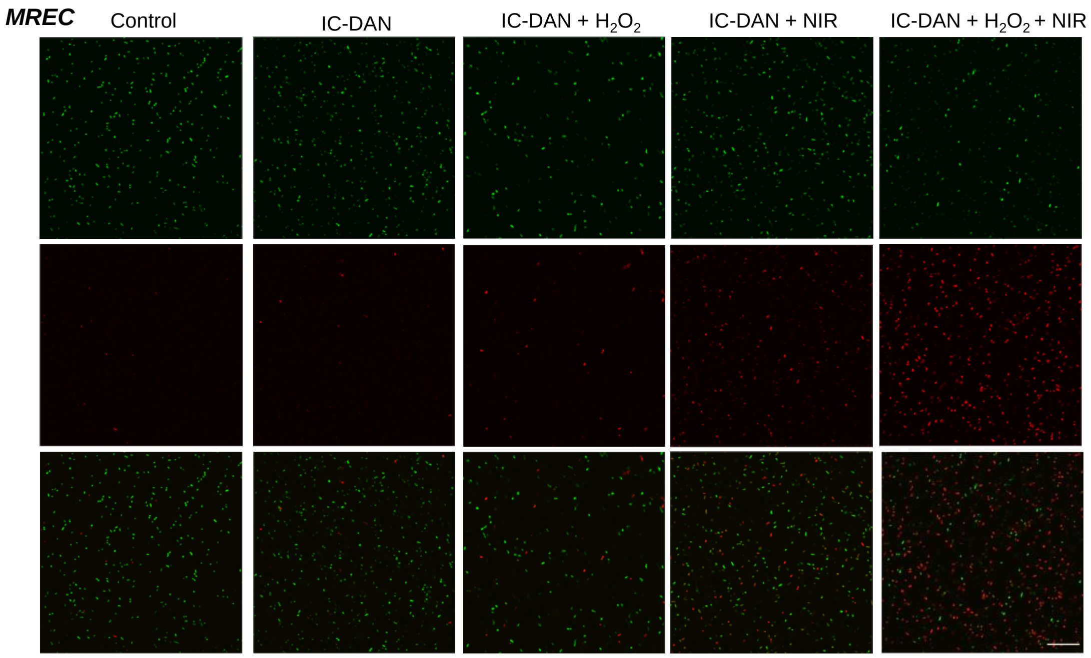

Figure S14. MREC cells upon varied treatment conditions.

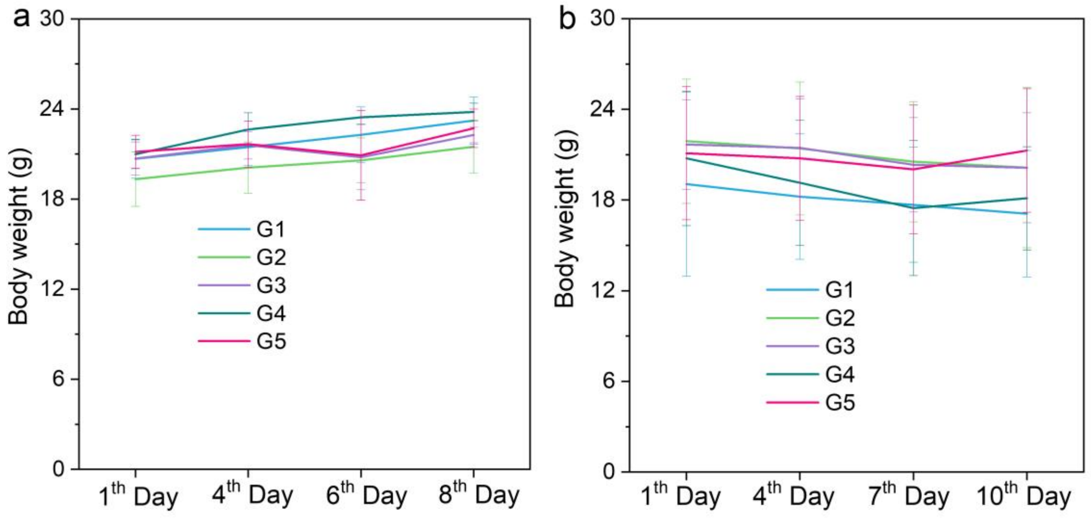

Figure S15. (a) body weight of infected wounds in normal mice and (b) type 2 diabetes mice under different treatments.

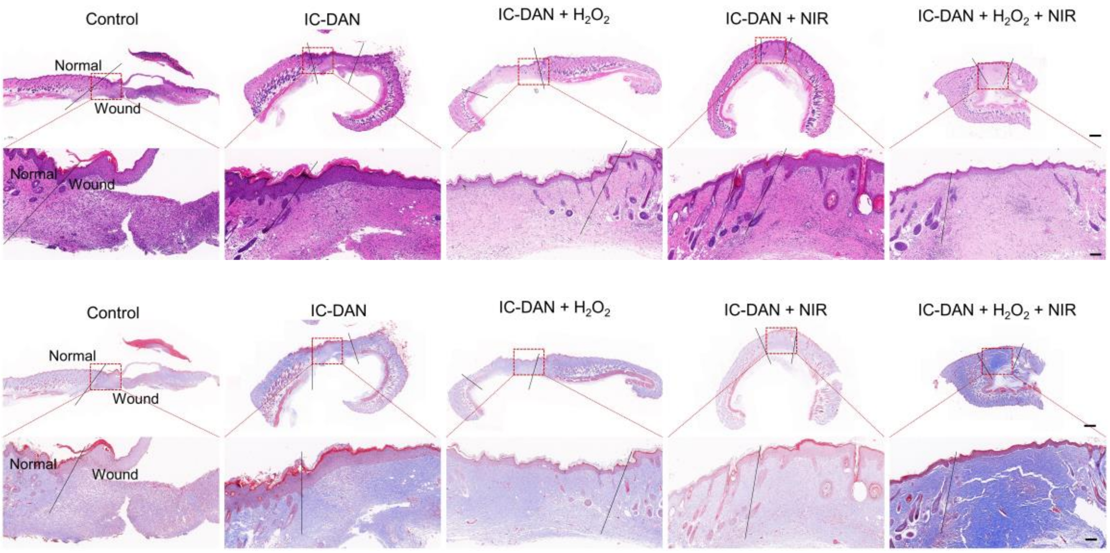

Figure S16. H&E and Masson staining of infected wounds in normal mice after different treatments (left: normal, right: wound, scale bar = 500 μm, 100 μm).

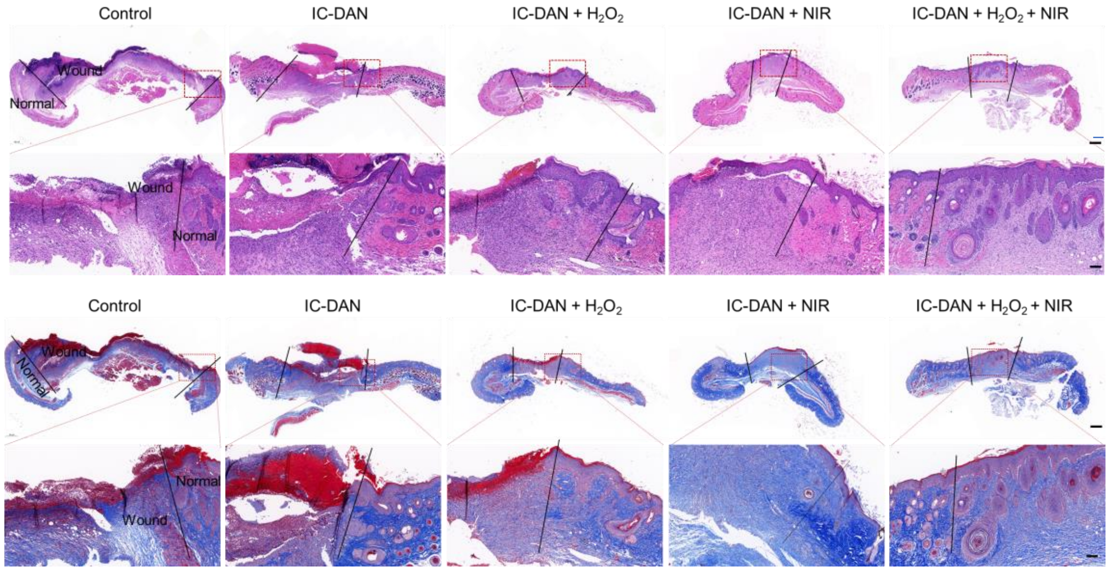

Figure S17. H&E and Masson staining of infected wounds in type 2 diabetes mice after different treatments (left: normal, right: wound, scale bar = 500 μm, 100 μm).

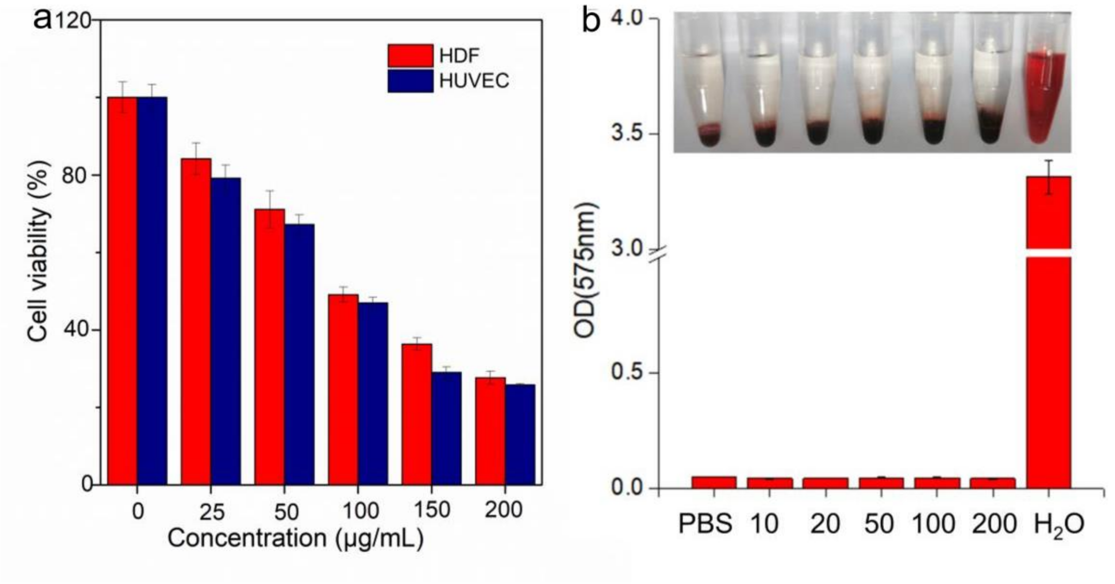

Figure S18. (A) The relative absorbance of HUVEC and HDF cells after being treated with IC-DAN at different concentrations for 24 h. (B) Hemolysis assessment induced by IC-DAN at various concentrations. Insert picture indicates the direct observation of hemolysis by IC-DAN.

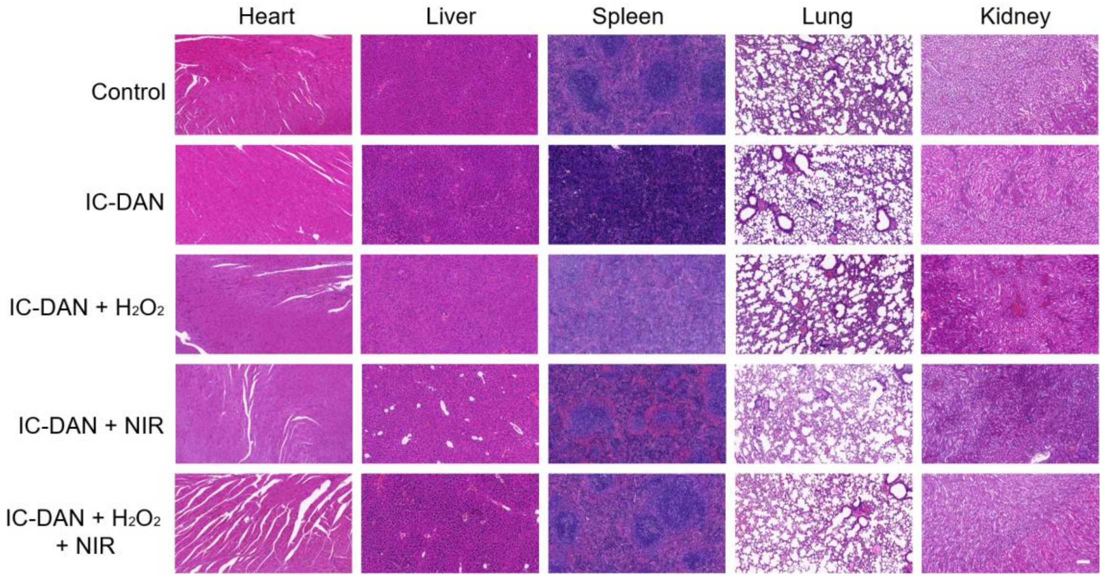

Figure S19. H&E staining of main organs (heart, liver, spleen, lung and kidney) of type 2 diabetes infection mice after different treatments (scale bar = 100 μm).

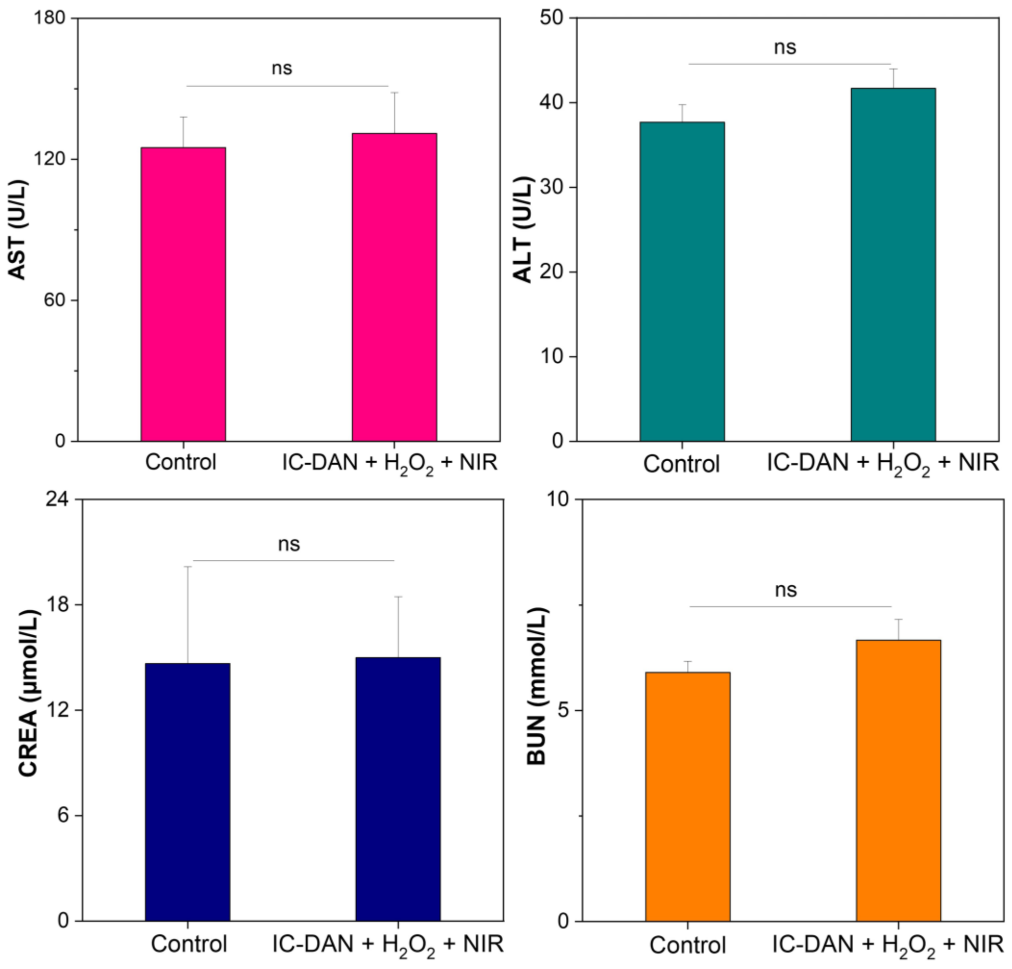

Figure S20. Biochemical results of normal infection mice after different treatments.

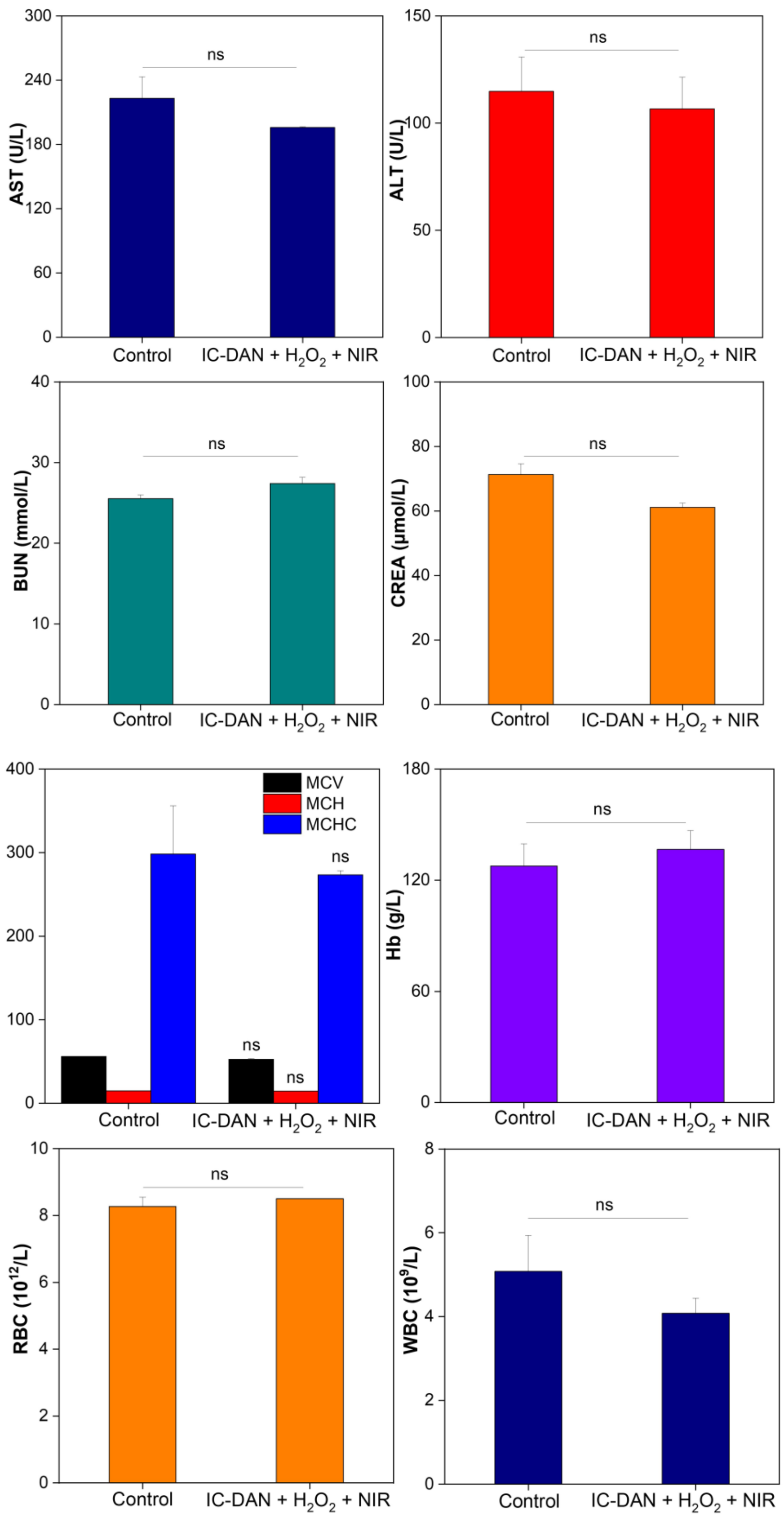

Figure S21. Blood routine test and biochemical results of type 2 diabetes infection mice after different treatments.

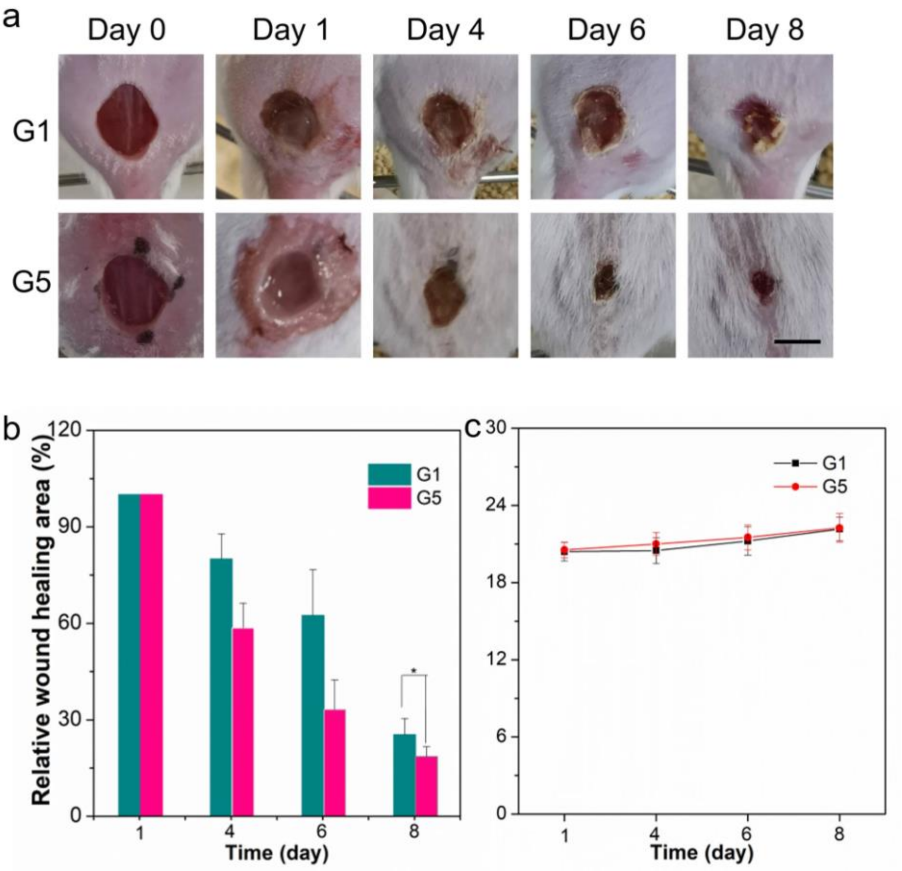

Figure S22. (a) corresponding digital photographs of mice in MREC-infected mice. Scale bar: 5mm. (b) Relative wound healing area in MREC-infected mice (n = 5). Values are presented as mean ± SD. (c) Body weight in MREC-infected mice (n = 5). Values are presented as mean ± SD. G1, control; G5, IC-DAN + H_2O_2 + NIR. *p < 0.05.

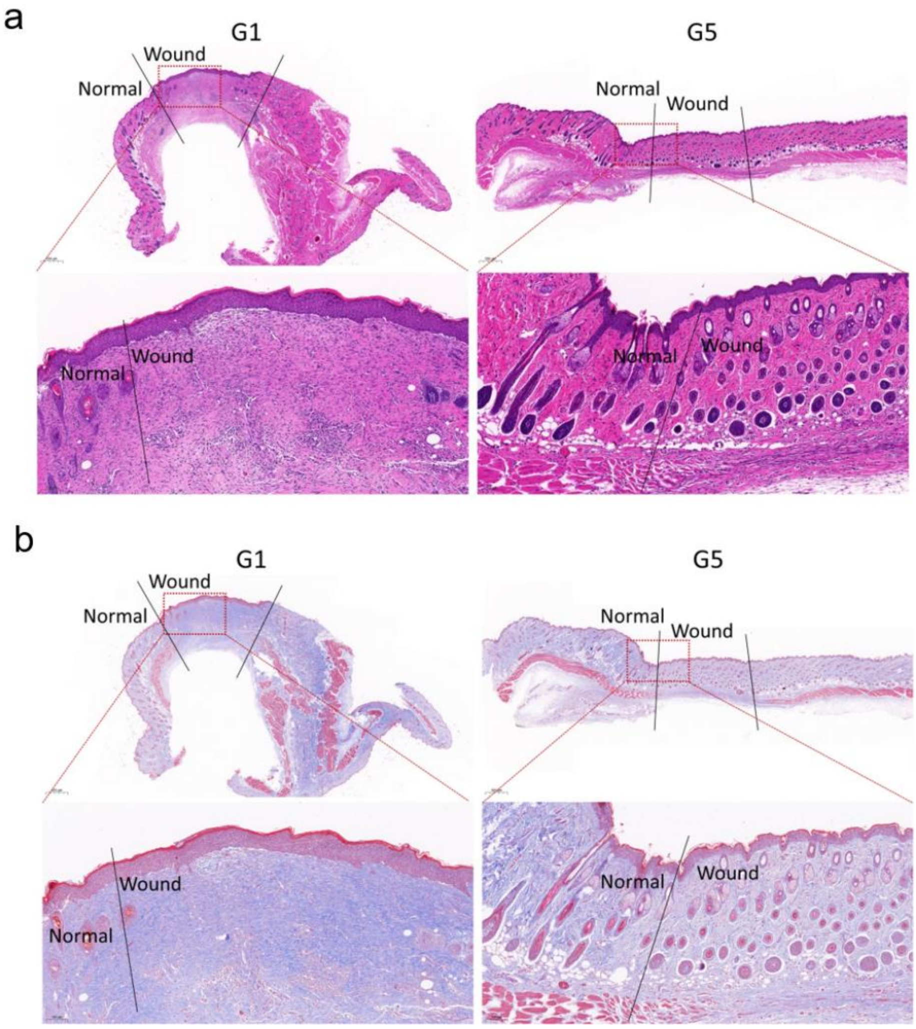

Figure S23. (a) H&E and Masson staining of infected wounds in MREC-infected mice after different treatments. (b) Masson staining of infected wounds in MREC-infected mice after different treatments (left: normal, right: wound, scale bar = 500 μm, 100 μm). G1, control; G5, IC-DAN + H_2O_2 + NIR. *p < 0.05.

Table S1. XPS and ICP-OES parameters of SAN and IC-DAN.

Sample	C wt%	N wt%	O wt%	Fe wt%	Fe ICP-OES
SAN	83.42	6.76	9.24	2.54	1.91 wt%
IC@DAN	81.84	6.53	10.87	3.30	2.84 wt%

Table S2. Structural parameters of N-CC@Fe SA, N-CC@Fe DA and Fe foil and extracted from the EXAFS fitting.

	Path	N	100xR(Å)	ΔE(eV)	10^3xσ^2(Å^2)	R-factor
SAN	Fe-N	4.50	2.02	0.82(0.33)	11.9(1.23)	0.001
IC-DAN	Fe-N	4.36	2.02	1.73(0.18)	10.0(1.85)	0.003
	Fe-Fe	0.80	2.72.	8.63(3.25)	2.65(0.59)	
	Fe-Fe	8	2.46			
Fe foil	Fe-Fe	6	2.83			

S_0^2, 0.97, is the amplitude reduction factor; N is the coordination number; R is the interatomic distance (the bond length between central atoms and surrounding coordination atoms), σ^2 is Debye-Waller factor (a measure of thermal and static disorder in absorber-scatter distances); ΔE is edge-energy shift (the difference between the zero kinetic energy value of the sample and that of the theoretical model). R factor is used to value the goodness of the fitting.

Table S3. Comparison of the kinetics for the SAN and IC-DAN toward H_2O_2.

	[E/Fe] (M)	K_m (mM)	V_{max} (M s^{-1})	K_{cat} (s^{-1})
SAN	4.75×10^{-7}	8×10	1.16×10^{-6}	2.44
IC@DAN	4.49×10^{-7}	2.37×10^{2}	2.21×10^{-6}	4.92

Table S4. Comparison of the kinetics for the SAN and IC-DAN toward TMB.

	[E/Fe] (M)	K_m (mM)	V_{max} (M s^{-1})	K_{cat} (s^{-1})
SAN	3×10^{-8}	0.08	0.86×10^{-6}	2.81
IC@DAN	2.36×10^{-8}	0.17	1.53×10^{-6}	6.49

Reference

1. Kresse, G. et al. Efficiency of ab-initio total energy calculations for metals and semiconductors using a plane-wave basis set. *Comput. Mater. Sci.* **1996**, *6*, 15-50.
2. Perdew, J. P. et al. Generalized Gradient Approximation Made Simple. *Phys. Rev. L.* **1996**, *77*(10), 3865-386.
3. Computational Chemistry Comparison and Benchmark Database. http://cccbdb.nist.gov/.
